# Acetyltransferase NAT10 promotes an immunosuppressive microenvironment by modulating CD8^+^ T cell activity in prostate cancer

**DOI:** 10.1186/s43556-024-00228-5

**Published:** 2024-12-09

**Authors:** Ji Liu, Zhuoran Gu, Libin Zou, Zhijin Zhang, Liliang Shen, Ruiliang Wang, Shaobo Xue, Jiang Geng, Shiyu Mao, Wentao Zhang, Xudong Yao

**Affiliations:** 1 Department of Urology, School of Medicine, Shanghai Tenth People’s Hospital, Tongji University, Shanghai, 200072 China; 2 Institute of Urinary Oncology, School of Medicine, Tongji University, Shanghai, 200092 China; 3 Department of Central Laboratory, Clinical Medicine Scientific and Technical Innovation Park, Shanghai Tenth People’s Hospital, Shanghai, 200435 China; 4 Department of Urology, the Affiliated People’s Hospital of Ningbo University, 251 East Baizhang Road, Ningbo City, Zhejiang Province 315040 China

**Keywords:** Prostate cancer, Ac4C acetylation, Immune microenvironment, Immunotherapy

## Abstract

N-acetyltransferase 10 (NAT10), an enzyme responsible for ac4C acetylation, is implicated in cancer progression, though its specific biological function in prostate cancer remains insufficiently understood. This study clarifies NAT10’s role in prostate cancer and its effects on the tumor immune microenvironment. NAT10 expression and clinical relevance were assessed through bioinformatics, RT-qPCR, and IHC analyses, comparing prostate cancer tissues with normal controls. The impact of NAT10 on tumor cell proliferation, migration, and invasion was investigated via in vitro assays—including CCK-8, EdU, wound healing, and 3D-Transwell—as well as in vivo mouse xenograft models and organoid studies. Further, NAT10’s influence on immune cell infiltration was examined using flow cytometry, IHC, cell co-culture assays, and ELISA to elucidate downstream chemokine effects, specifically targeting CD8^+^ T cells. Findings indicated significant upregulation of NAT10 in prostate cancer cells, enhancing their proliferative and invasive capacities. Notably, NAT10 suppresses CD8^+^ T cell recruitment and cytotoxicity through the CCL25/CCR9 axis, fostering an immunosuppressive microenvironment that exacerbates tumor progression. An ac4C modification score was also devised based on NAT10’s downstream targets, providing a novel predictive tool for evaluating immune infiltration and forecasting immunotherapy responses in patients with prostate cancer. This study underscores NAT10’s pivotal role in modulating the prostate cancer immune microenvironment, offering insights into the immune desert phenomenon and identifying NAT10 as a promising therapeutic target for improving immunotherapy efficacy.

**Supplementary Information:**

The online version contains supplementary material available at 10.1186/s43556-024-00228-5.

## Introduction

Prostate cancer (PCa) is the second most common cancer globally, with the highest incidence rates observed in North America, South America, Europe, Australia, and the Caribbean [1, 2]. The primary treatment for locally advanced and metastatic PCa is androgen deprivation therapy (ADT). Although ADT initially achieves tumor control in most patients, its effects typically last 18 to 24 months, after which ADT-resistant adaptations emerge, progressing the disease to castration-resistant prostate cancer (CRPC), often accompanied by bone or distant organ metastases [3]. For patients with metastatic CRPC, prognosis remains poor, with survival rarely exceeding 19 months. CRPC pathogenesis is driven by DNA and chromatin methylation, histone modifications that impair androgen receptor (AR) DNA binding, and AR-specific post-translational modifications such as phosphorylation, acetylation, methylation, ubiquitination, and ubiquitination-like changes, which enhance AR sensitivity to low androgen levels. These epigenetic alterations accelerate CRPC progression [4], underscoring the importance of epigenetics in understanding CRPC development and therapy.

Epigenetic RNA modifications represent a vital area within epigenetics, exerting substantial influence on cellular biology by modulating RNA production, transport, metabolism, and translation efficiency. Major RNA modifications include N6-methyladenosine (m6A), 5-methylcytosine (m5C), 7-methylguanosine (m7G), and N4-acetylcytidine (ac4C), which are emerging as essential post-transcriptional regulatory mechanisms across various physiological and pathological contexts [5, 6]. Among these, ac4C, the first discovered acetylated nucleoside, is widely present on mRNA, following m6A, m5C, and m7G in abundance [7, 8]. Initially identified within the bacterial tRNA anticodon, ac4C is also found in eukaryotic cell serine and leucine tRNAs and 18S rRNA [9]. Arango et al. identified multiple acetylation sites within the human transcriptome, such as ac4C [7]. They observed that mRNA acetylation within coding sequences enhances mRNA stability and translation, while ac4C acetylation in the wobble checkpoint promotes translation efficiency [7]. Recent findings indicate that NAT10 or its homologs catalyze ac4C modifications across mRNA, tRNA, and 18S RNA in various species [10]. Additionally, mRNA ac4C production, catalyzed by acetylation processes, regulates target gene expression, thereby contributing to the development of various cancers, including bladder, gastric, colon, and cervical cancers. Nonetheless, limited investigation into NAT10’s biological functions in PCa progression positions it as a promising candidate for future diagnostic and therapeutic exploration in this malignancy [11–14].

Recent research demonstrates that acetylation facilitates mRNA ac4C synthesis, regulating target genes and promoting the progression of various cancers. Wang et al. identified that NAT10-mediated acetylation modifications enhance cancer cell proliferation, migration, and stemness in bladder cancer through the ac4C modification of SOX4 and AKT1 [12]. Similarly, Deng et al. found that NAT10-mediated ac4C acetylation of MDM2 stabilizes its mRNA, leading to MDM2 upregulation, which negatively regulates p53, thereby advancing gastric carcinogenesis [14]. Li et al. reported that NAT10 promotes PCa cell proliferation and epithelial-to-mesenchymal transition (EMT) by acetylating HMGA1 and KRT8 mRNAs [15]. Despite these findings, the role of NAT10-mediated ac4C acetylation in PCa remains inadequately understood, necessitating a deeper investigation into its impact on PCa biological behaviors and mechanisms.

Current treatments for metastatic CRPC (mCRPC), including ADT, chemotherapy, PARP inhibitors, and immunotherapy, have limited efficacy, with chemotherapy and immunotherapy extending median survival by only around four months [16]. Recently, immune checkpoint inhibitors targeting CTLA-4 and PD-1/PD-L1 have shown promise in solid and hematological tumors by blocking co-inhibitory signals and enhancing CD8^+^ T cell-mediated tumor destruction [17]. However, the response rate to these therapies in advanced PCa remains low, approximately 5% [18]. A primary challenge is the insufficient infiltration of activated CD8^+^ T cells within the tumor microenvironment (TME), a key factor limiting immunotherapy success [19]. Within the TME, activated CD8^+^ T cells are critical for tumor cell eradication, releasing perforin, Fas, and cytokines such as TNF-α and IFN-γ, which exert anti-tumor effects [20]. Aberrant epigenetic regulation disrupts core gene expression in tumor cells, enabling immune evasion within the TME and promoting tumor progression. This study provides a theoretical basis for the current research [21].

This study presents an in-depth analysis of ac4C acetyltransferase NAT10’s pivotal role in promoting an immune desert phenotype in PCa. By regulating EMT pathway markers and suppressing CD8^+^ T cell recruitment and cytotoxic activity, NAT10 fosters an immunosuppressive TME, thereby accelerating tumor progression. Furthermore, this study developed an innovative ac4C modification score, a predictive tool that accurately assesses immune infiltration and evaluates immunotherapy efficacy in PCa, holding significant clinical applicability. This research introduces a promising avenue for novel immunotherapeutic targets, particularly in the context of CRPC.

## Results

### High expression of NAT10 in mCRPC is associated with and drives malignant progression of PCa in* vivo* and *vitro*

**Fig. 1:**
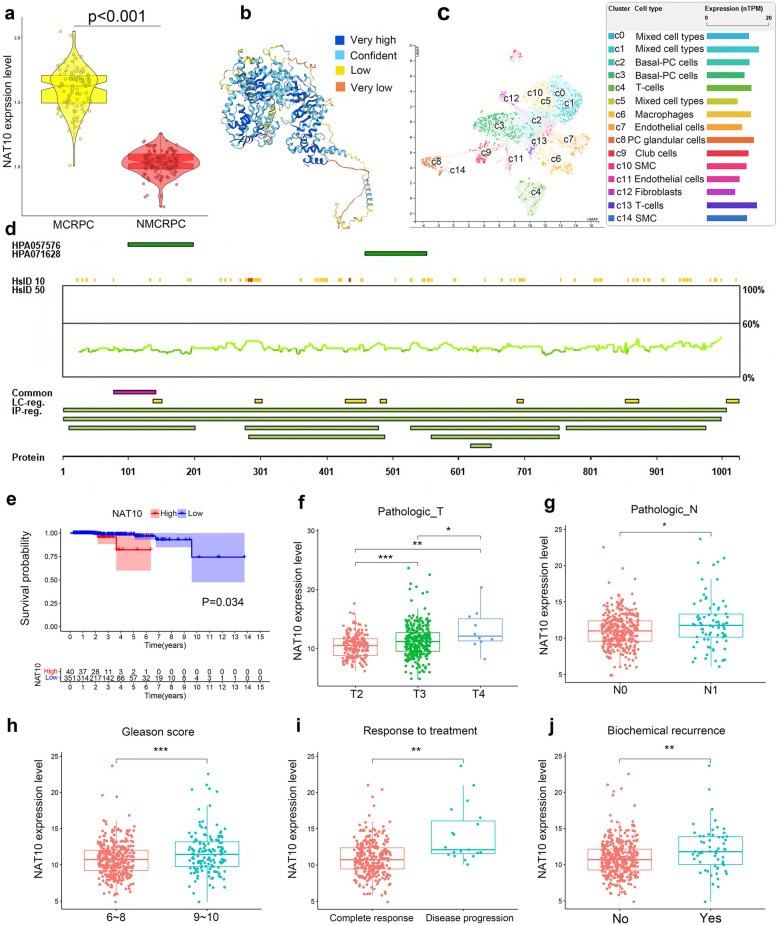
Significantly high expression of NAT10 in mCRPC is associated with prostate malignancy progression. **a** Expression levels of NAT10 in nmCRPC versus mCRPC samples (*p* < 0.001). **b** Protein three-dimensional structure of NAT10. **c** Expression levels of NAT10 in prostate single-cell sequencing. **d** Protein profile of NAT10. **e** Kaplan–Meier curves showed a significant negative correlation between NAT10 and the patient’s PFI. **f**-**j** Differential Analysis of NAT10 in Prostate Cancer Clinical Characteristics Subgroups

Box plot analysis revealed a statistically significant overexpression of NAT10 in mCRPC relative to non-metastatic CRPC (nmCRPC) (Fig. 1a). To elucidate its structural and functional domains, the three-dimensional structure of the NAT10 protein was analyzed (Fig. 1b). Single-cell sequencing was conducted to investigate cell-specific expression within the prostate, identifying NAT10’s predominant expression in glandular epithelial cells, T cells, and macrophages, suggesting its involvement in key cellular functions (Fig. 1c). Further profiling of NAT10 expression patterns was presented via protein profile data (Fig. 1d). Survival analysis using The Cancer Genome Atlas-Prostate Adenocarcinoma (TCGA-PRAD) data indicated a significant inverse correlation between NAT10 expression and progression-free survival, with higher NAT10 levels linked to poorer outcomes (Fig. 1e). Additionally, box plot analysis revealed statistically significant positive correlations between NAT10 expression and clinical indicators, including pathological T-stage, N-stage, and Gleason score, as well as associations with biochemical recurrence and drug resistance in patients with PCa (Fig. 1f-j). These results collectively highlight NAT10’s potential role as a prognostic biomarker for PCa progression.

**Fig. 2:**
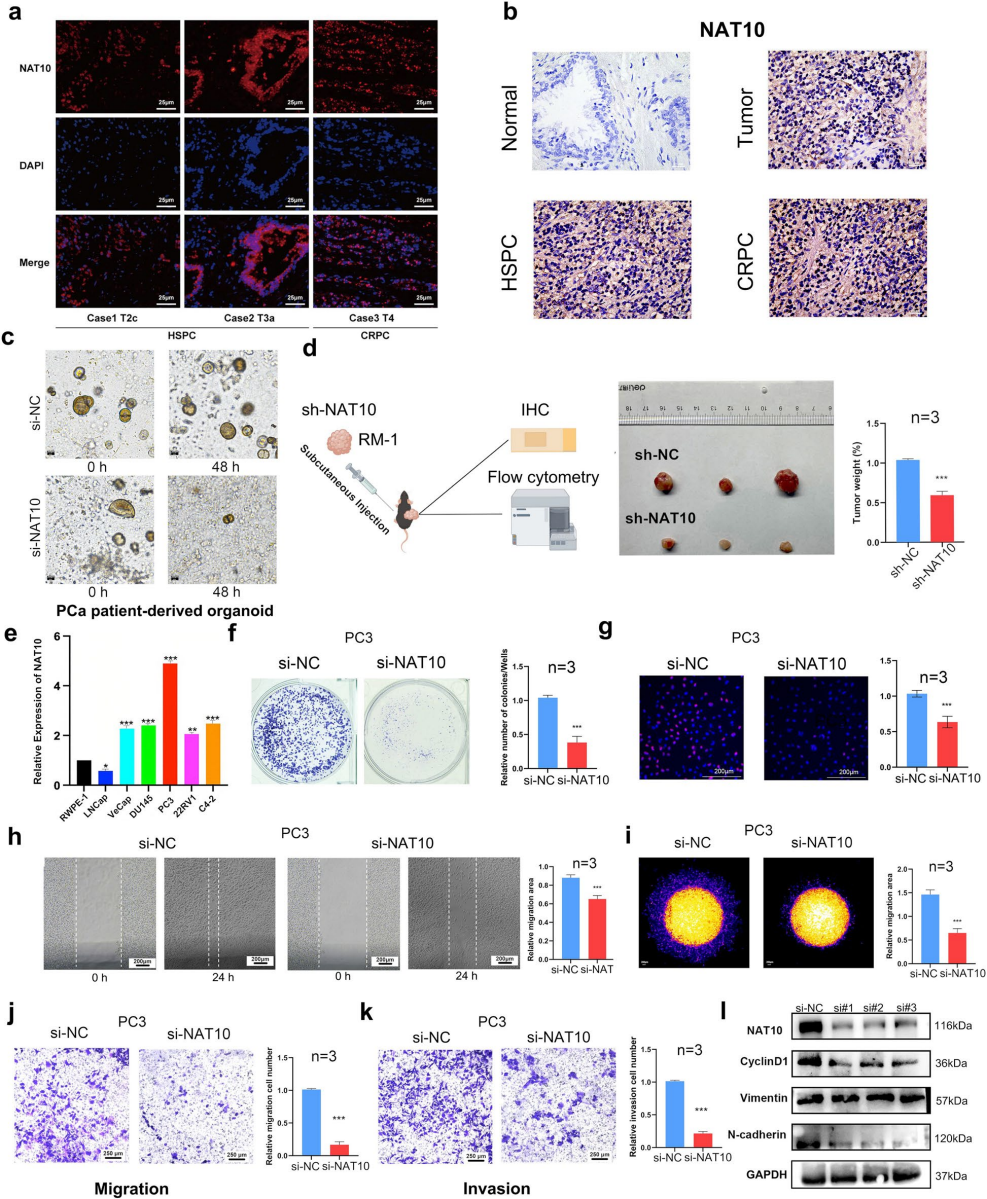
NAT10 levels in vitro and in vivo affect malignant progression of prostate cancer. **a** Immunofluorescence results showing NAT10 expression in different prostate cancer patient tissues. **b** IHC results showing NAT10 expression in tissues of different prostate cancer patients. **c** Organoid assay demonstrating prostate cancer organoid survival 48 h after addition of si-NAT10. **d** A mouse subcutaneous tumor formation assay demonstrated a significant decrease in the proliferative capacity of prostate cancer cells after knockdown of NAT10 at the in vivo level. **e** Bar graph showing transcript levels of NAT10 in prostate normal cell lines (RWPE-1) versus prostate cancer cell lines (VCaP, C4-2, PC3, DU145, LNCaP, and 22RV1). **f**-**g** Plate cloning and EdU assay demonstrates changes in cell proliferation capacity after knockdown of NAT10. **h**–**k** 3D-Transwell, Wounding-healing and Transwell assay demonstrated the changes in cell invasion and migration ability after knockdown of NAT10. (l) WB experiments demonstrated changes in the expression of proliferation- and invasion-related proteins after knockdown of NAT10. p value calculated using the Wilcoxon-test. (*p < 0.05; **p < 0.01; ***p < 0.001)

IF results of patient-derived PCa tissues confirmed NAT10’s nuclear localization, with higher expression in CRPC samples (Fig. 2a-b). In PCa patient-derived organoid, NAT10 knockdown also significantly inhibited PCa growth (Fig. 2c). In the in vivo part of the experiment, RT-qPCR was used to validate the knockdown efficiency of three mouse sh-Nat10 constructs, and sh-Nat10#3 was selected for further studies (Fig. S1a). Subcutaneous tumor formation experiments in nude mice indicated that knockdown of Nat10 significantly inhibited tumor growth (Fig. 2d). In vitro expreriment,the relative expression of NAT10 in various PCa cell lines (RWPE-1, VCaP, C4-2, PC3, DU145, LNCaP, and 22RV1) was assessed via RT-qPCR (Fig. 2e). RT-qPCR was used to validate the knockdown efficiency of three si-NAT10 constructs, and si-NAT10#2 was selected for subsequent studies (Fig. S1b-c). To explore NAT10's role in CRPC progression, functional assays (plate cloning, EdU, and CCK-8) conducted post-NAT10 knockdown showed significant inhibition of CRPC cell proliferation (Fig. 2f-g, S1d). Additionally, 3D Transwell, Transwell and wound-healing assays indicated that NAT10 knockdown effectively suppressed CRPC cell invasion and migration (Fig. 2h-k). Further validation in DU145 cells through Transwell, and colony formation assays (Fig. S2). Western blot analysis showed reduced levels of proliferation marker (CyclinD1) and EMT marker (N-cadherin,Vimentin) post-NAT10 knockdown (Fig. 2l). These findings suggest that NAT10 promotes PCa cell proliferation, invasion, and migration.

### NAT10 exhibits a regulatory association with immune cells within the immune microenvironment

**Fig. 3:**
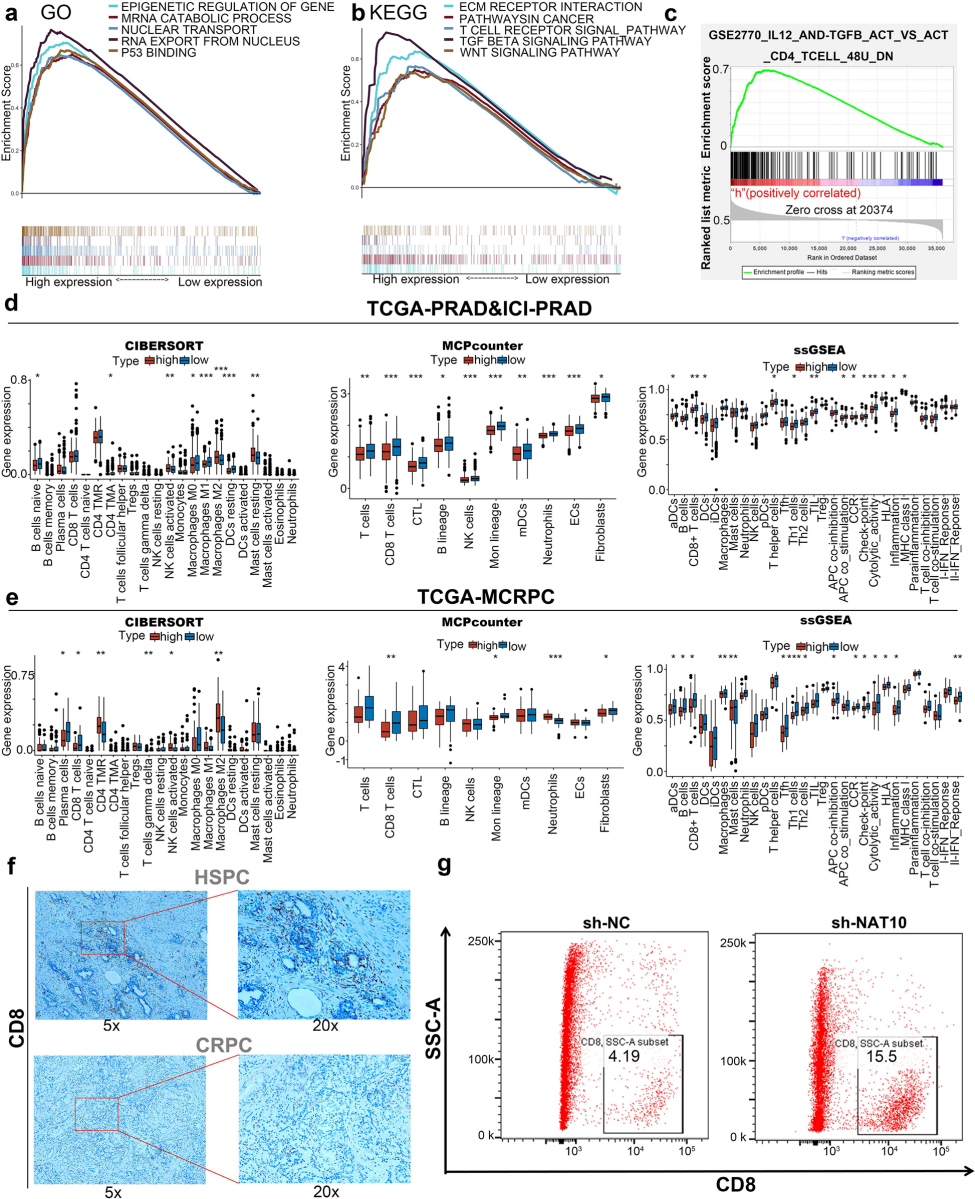
NAT10 exhibits a regulatory association with immune cells within the immune microenvironment. **a**-**c** GSEA demonstrates the possible functions of NAT10, and the pathways involved. **d**-**e** Evaluation of immune cell infiltration in TGCA-PRAD, ICGC-PRAD, and TCGA-MCRPC datasets by CIBERSORT, MCPcounter, and ssGSEA algorithms. **f** IHC demonstrates the expression of CD8 in CRPC and HSPC samples. **g** Correlation of NAT10 with CD8^+^T cells explored by flow cytometry. (*p < 0.05; **p < 0.01; ***p < 0.001)

To elucidate the functional roles of NAT10 in PCa, GSEA analysis identified the potential involvement of NAT10 in nuclear translocation, RNA nuclear export, RNA metabolism, and EMT-related pathways, including stem cell pathways (Wnt and TGF-β), Extracellular Matrix (ECM) receptor pathways, and T cell receptor pathways (Fig. 3a-b). To examine the relationship between NAT10 and local immune cell infiltration, immune-related signatures were applied, revealing a significant association between NAT10 expression and immune cell infiltration (Fig. 3c). Using the TCGA-PRAD and ICGC-PRAD PCa datasets, immune cell infiltration was further analyzed through CIBERSORT, MCPcounter, and ssGSEA algorithms. CIBERSORT analysis indicated a significant correlation between NAT10 expression and various immune cell populations, while MCPcounter analysis showed a significant negative correlation between NAT10 expression and CD8^+^ T cells, virulent lymphocytes, NK cells, and monocytes. ssGSEA results corroborated this, revealing a negative association with immune responses, including CCR and immune checkpoint activation (Fig. 3d). Analysis of TCGA-MCRPC samples supported these findings, suggesting a role for NAT10 in immune modulation (Fig. 3e). IHC analysis of human HSPC and CRPC samples revealed a significant downregulation of CD8 expression in CRPC samples, indicating the presence of an immunosuppressive microenvironment in these tumors (Fig. 3f). Flow cytometry showed an inverse correlation between NAT10 expression and CD8^+^ T cell infiltration in xenograft tumor model, consistent with public database analyses (Fig. 3g). These results indicate that NAT10 may regulate immune cell infiltration, particularly CD8^+^ T cells, thereby contributing to an immunosuppressive microenvironment in PCa.

### NAT10 fosters a PCa-suppressive immune microenvironment by modulating the CCL25/CCR9 *axis*

**Fig. 4:**
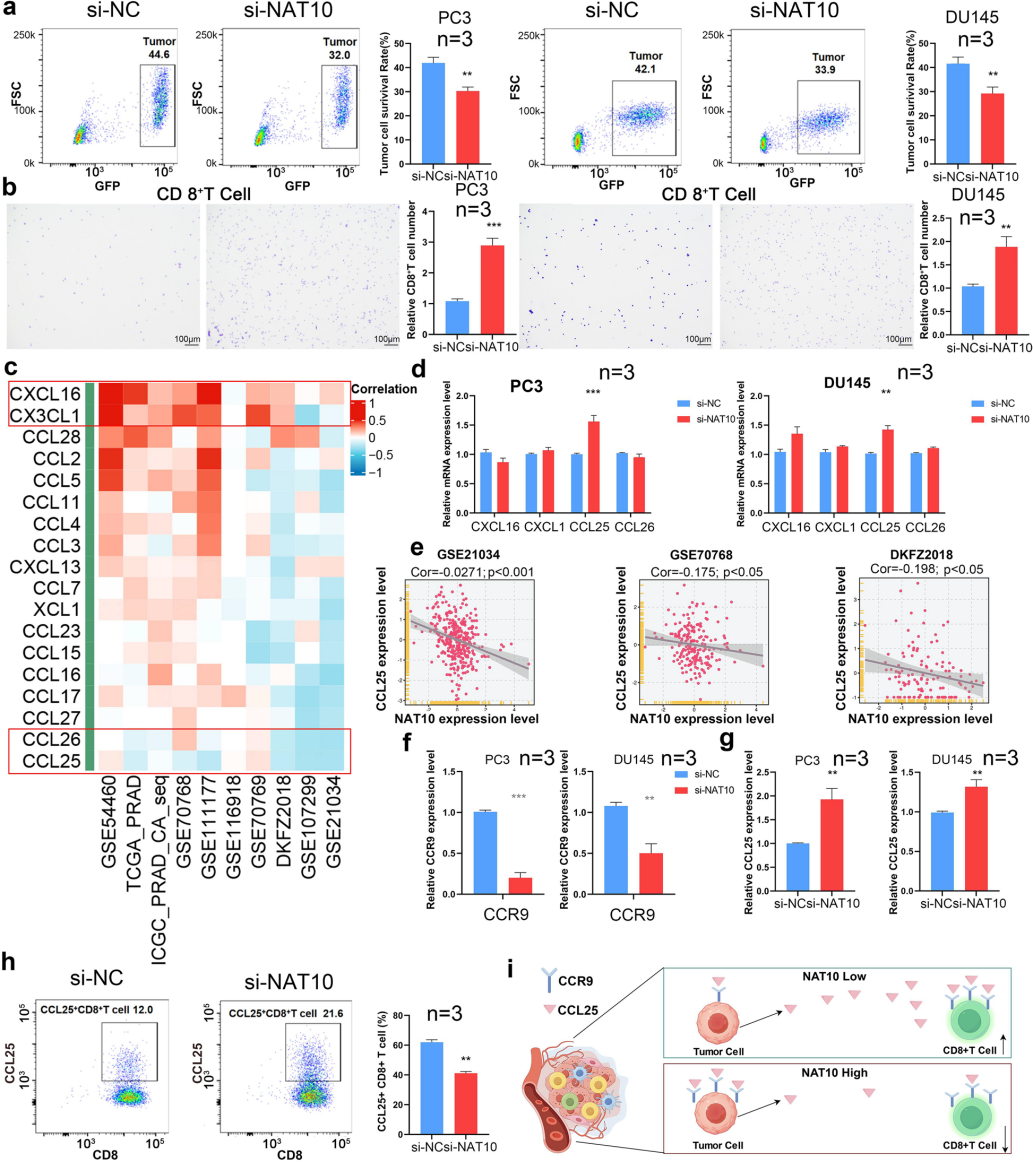
NAT10 fosters a prostate cancer-suppressive immune microenvironment by modulating the CCL25/CCR9 axis. **a** The tumor-killing capacity of CD8^+^ T cells was assessed by flow cytometry after co-culturing CD8^+^ T cells with tumor cells. **b** The recruitment of tumor cells to CD8^+^ T cells was assessed using a Transwell assay, with tumor cells placed in the lower chamber and immune cells in the upper chamber. **c**-**e** Chemokines regulated downstream of NAT10 were screened using the Best database and subsequently validated through RT-qPCR experiments. **f**–**h** The changes in CCL25 and its receptor CCR9 in tumor cells after NAT10 knockdown were elucidated using RT-qPCR, ELISA, and flow cytometry assays. **i** Hypothesized mechanism of NAT10 Regulation of CD8^+^ T Cell infiltration via the CCL25/CCR9 Axis. (*p < 0.05; **p < 0.01; ***p < 0.001)

To investigate how NAT10 regulates CD8^+^ T cell recruitment and cytotoxicity, co-culture experiments assessed CD8^+^ T cell-mediated killing of CRPC cells. NAT10 knockdown significantly impaired CD8^+^ T cell cytotoxicity at a 1:1 tumor-to-CD8^+^ T cell ratio (Fig. 4a). Transwell migration assays showed that NAT10 knockdown markedly reduced CD8^+^ T cell migration toward CRPC cells in co-culture (Fig. 4b), suggesting that NAT10 limits CD8^+^ T cell recruitment and cytotoxicity in the tumor microenvironment. Bioinformatics analysis of public databases identified chemokines associated with NAT10: CXCL16 and CX3CL1 positively correlated, while CCL26 and CCL25 negatively correlated (Fig. 4c). RT-qPCR validation in CRPC cell lines (PC3 and DU145) after NAT10 knockdown showed a significant increase in CCL25 mRNA, aligning with predictions, leading to the selection of CCL25 for further study (Fig. 4d-e). Since CCR9 is the receptor for CCL25, CCR9 mRNA levels were also analyzed, revealing a marked decrease post-NAT10 knockdown (Fig. 4f). ELISA and flow cytometry confirmed that NAT10 knockdown significantly increased CCL25 levels, promoting CD8^+^ T cell recruitment and enhanced cytotoxicity in the tumor microenvironment (Fig. 4g-h). These findings suggest that NAT10 downregulates CCL25 in PCa cells while upregulating its receptor, CCR9, likely disrupting CCL25-CCR9 binding on CD8^+^ T cells. This mechanism may reduce CD8^+^ T cell infiltration and impair cytotoxic function (Fig. 4i).

### Establishment and analysis of tumor immune microenvironment scores and identification of ac4C-related genes associated with malignant progression in PCa

**Fig. 5:**
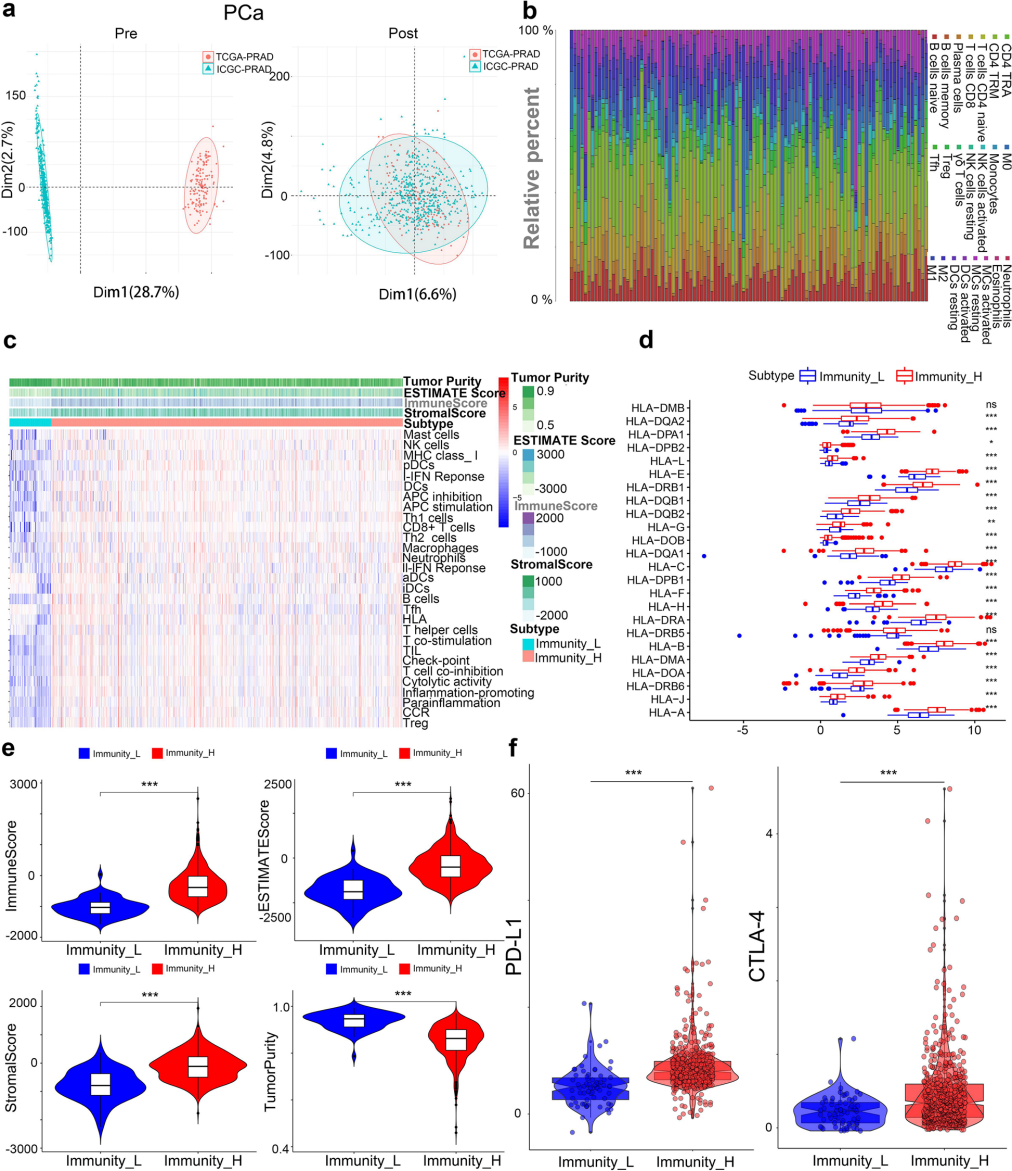
Establishment and analysis of tumor immune microenvironment scores in PCa. **a** Samples from TCGA-PARD and ICGC-PRAD were integrated by a debatch function and demonstrated using Principal Component Analysis (PCA). **b** Bar graph showing the distribution of TCGA-PRAD versus ICGC-PRAD immune cells. **c** Tumor immune cell infiltration between different subgroups based on tumor microenvironment score. **d** Expression of HLA-related genes in high versus low immune score groups. **e** Expression levels of immune, stromal, tumor purity and microenvironmental scores in the high and low immune score groups. **f** Expression of PD-L1 and CTLA-4 in high versus low immune score groups. (*p < 0.05; **p < 0.01; ***p < 0.001)

**Fig. 6:**
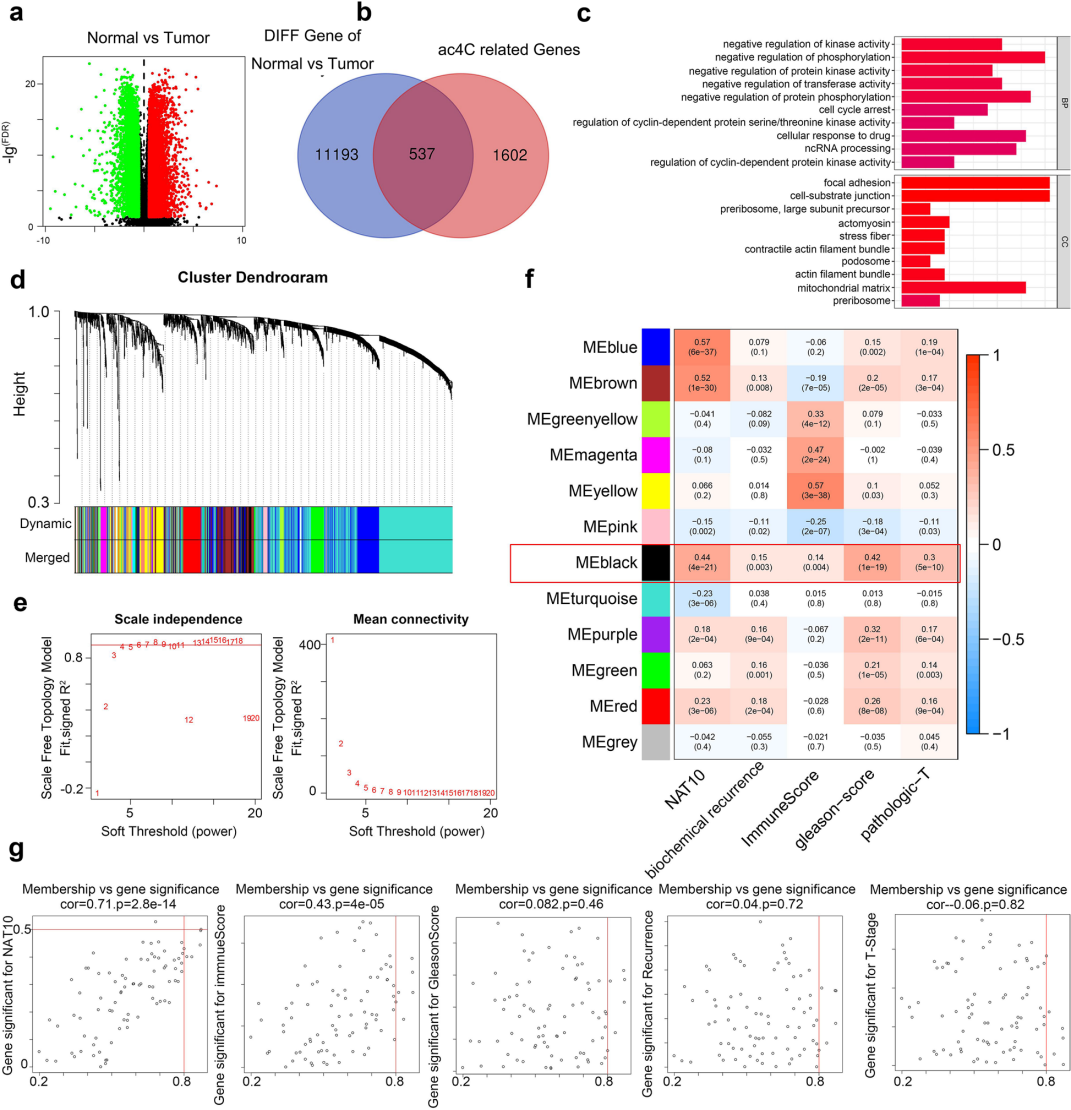
Identification of genes associated with ac4C, tumor immune microenvironment and malignant progression in CRPC. **a** Volcano diagram showing differential genes between normal and tumor tissues in the prostate. **b** Wayne diagram showing intersecting genes of DEGs with ac4C-related genes. **c** GO analysis demonstrates possible functions of intersecting genes. **d**-**f** The correlation of different modules with NAT10, the tumor immune microenvironment score, and the clinical factors was analyzed by clustering the genes and after dimensionality reduction. **g** Correlation of genes in the analysis module (**p < 0.01; ***p < 0.001)

To ensure data consistency, the TCGA-PRAD and ICGC-PRAD datasets were de-batched, confirming proper alignment post-de-batching (Fig. 5a). Immune cell infiltration in 639 PCa samples was assessed via ssGSEA, followed by unsupervised clustering to classify samples into high and low immune infiltration groups (Fig. 5b). The ESTIMATE algorithm then calculated estimated scores, tumor purity, stromal scores, and immune scores. The heatmap showed that high immune infiltration correlated with increased immune scores and reduced tumor purity (Fig. 5c). For further analysis, samples were divided into immunity_H and immunity_L groups by immune scores, revealing significant differences in HLA-related gene expression (*p* < 0.001) (Fig. 5d). There are significant differences in scores across different immune groups, and immunotherapy-related genes, including PD-L1, CTLA-4, also showed significant differential expression between groups (Fig. 5e-f). Differential expression analysis normal and tumor samples identified 11,730 genes with significant differences (FDR < 0.05, |LogFC|> 1) (Fig. 6a). NAT10-mediated ac4C modifications were linked to 2,156 genes, with 537 ac4C-related genes showing differential expression between normal and tumor (Fig. 6b). Gene Ontology (GO) analysis of these DEGs indicated roles in kinase regulation and cell adhesion, suggesting functional relevance in tumor biology (Fig. 6c). Weighted gene co-expression network analysis (WGCNA) analysis, applied to 639 PCa samples and 537 ac4C-associated different expression genes (DEGs), grouped these genes into a 12-gene module, with β = 5 as the optimal soft threshold (Fig. 6d-e).To investigate clinical relevance, the MEBlack module genes were analyzed for correlations with NAT10 expression, immune microenvironment scores, and clinical factors such as Gleason score, T-stage, and biochemical recurrence (*p* < 0.05) (Fig. 6f). Validation of inter-gene correlations within the module reinforced their relevance to PCa progression (Fig. 6g). These findings suggest that NAT10 may regulate gene expression through ac4C modifications, fostering an immunosuppressive microenvironment in PCa, and highlight immune-prognostic genes with potential roles in PCa progression.

### Developing the ac4C-related subtypes

**Fig. 7:**
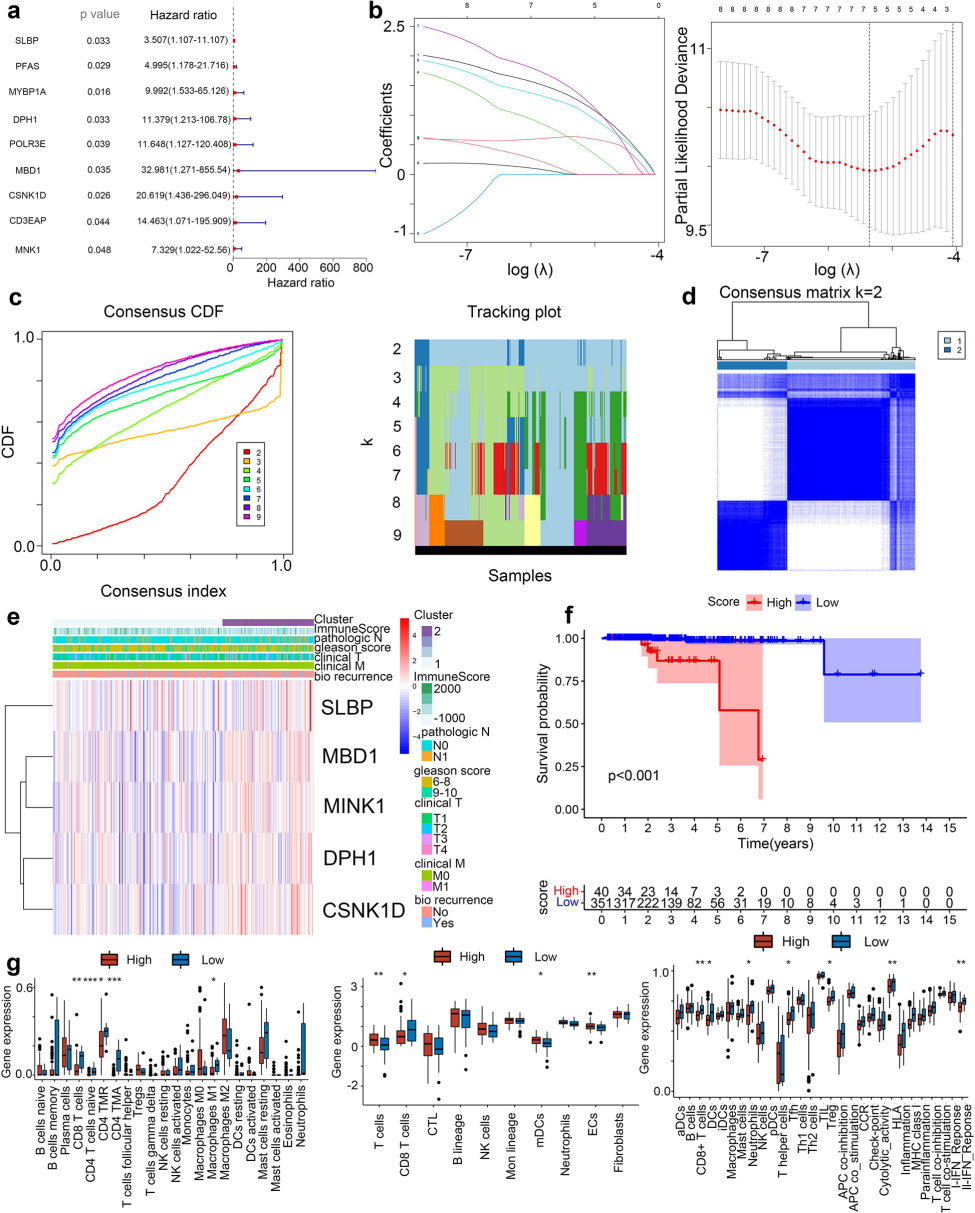
Developing the ac4C-related subtype. **a**-**b** Genes associated with prognosis in the module were screened by Unicox analysis and de-fitted by Lasso regression analysis. **c**-**d** Clustering dendrogram of PCa samples and genes clustering results. **e** Heat map showing the correlation between Clinicopathological features and expression of principal component genes. **f** Survival curves show prognostic differences between subtypes. **g** Box line plot showing differences in tumor-infiltrating immune cell between subtypes. (*p < 0.05; **p < 0.01; ***p < 0.001; #*p* > 0.05)

To identify genes within the MEBlack module associated with progression-free interval (PFI), UniCOX analysis identified nine genes with significant negative correlations to PFI, qualifying them for further investigation (Fig. 7a). These genes were refined via Lasso analysis, resulting in the selection of five key genes—SLBP, MBD1, MINK1, DPH1, and CSNK1D—as classification factors (Fig. 7b). Using these genes, the TCGA-PRAD and ICGC-PRAD cohorts were re-clustered through Consensus Clustering, with k = 2 as the optimal subgroup number, establishing robust ac4C-based classification groups (Fig. 7c-d). A heatmap was generated to illustrate correlations between the identified ac4C subtypes and clinical characteristics, highlighting subtype-specific patterns (Fig. 7e). PCA was applied to convert ac4C subtypes into ac4C scores. For clinical relevance, these ac4C subtypes were further divided into High ac4C and Low ac4C clusters based on their ac4C scores. Kaplan–Meier analysis of clinical prognosis showed that patients within the High ac4C cluster had significantly worse outcomes than those in the Low ac4C cluster (*p* < 0.001) (Fig. 7f). To further characterize the immune landscape within these clusters, tumor-infiltrating immune cell levels in the High and Low ac4C clusters were assessed using ssGSEA, MCPcounter, and CIBERSORT algorithms. Findings indicated that the Low ac4C cluster exhibited significantly higher levels of tumor-infiltrating immune cell, including CD4, CD8, and macrophages, along with elevated immune function activation (Fig. 7g). To examine the role of NAT10 in tumor stem cell dynamics, the mRNAsi score—an index of sample dedifferentiation—was introduced. Correlation analyses explored relationships between the ac4C score and mRNAsi, as well as among NAT10, mRNAsi, the ac4C score, and principal component genes (Fig. S3). Results demonstrated a strong correlation between NAT10 and mRNAsi, indicating that NAT10 may play a role in modulating tumor stem cell behavior. Collectively, these results highlight essential genes for constructing an ac4C-based classification, showing that the High ac4C subtype correlates with poorer prognosis and reduced immune infiltration. Additionally, the strong association between NAT10 and tumor stem cell dedifferentiation underscores its potential importance in PCa progression.

### Validation of principal component genes within subtypes from the cellular level and prediction of drugs sensitive to ac4C subtypes

**Fig. 8:**
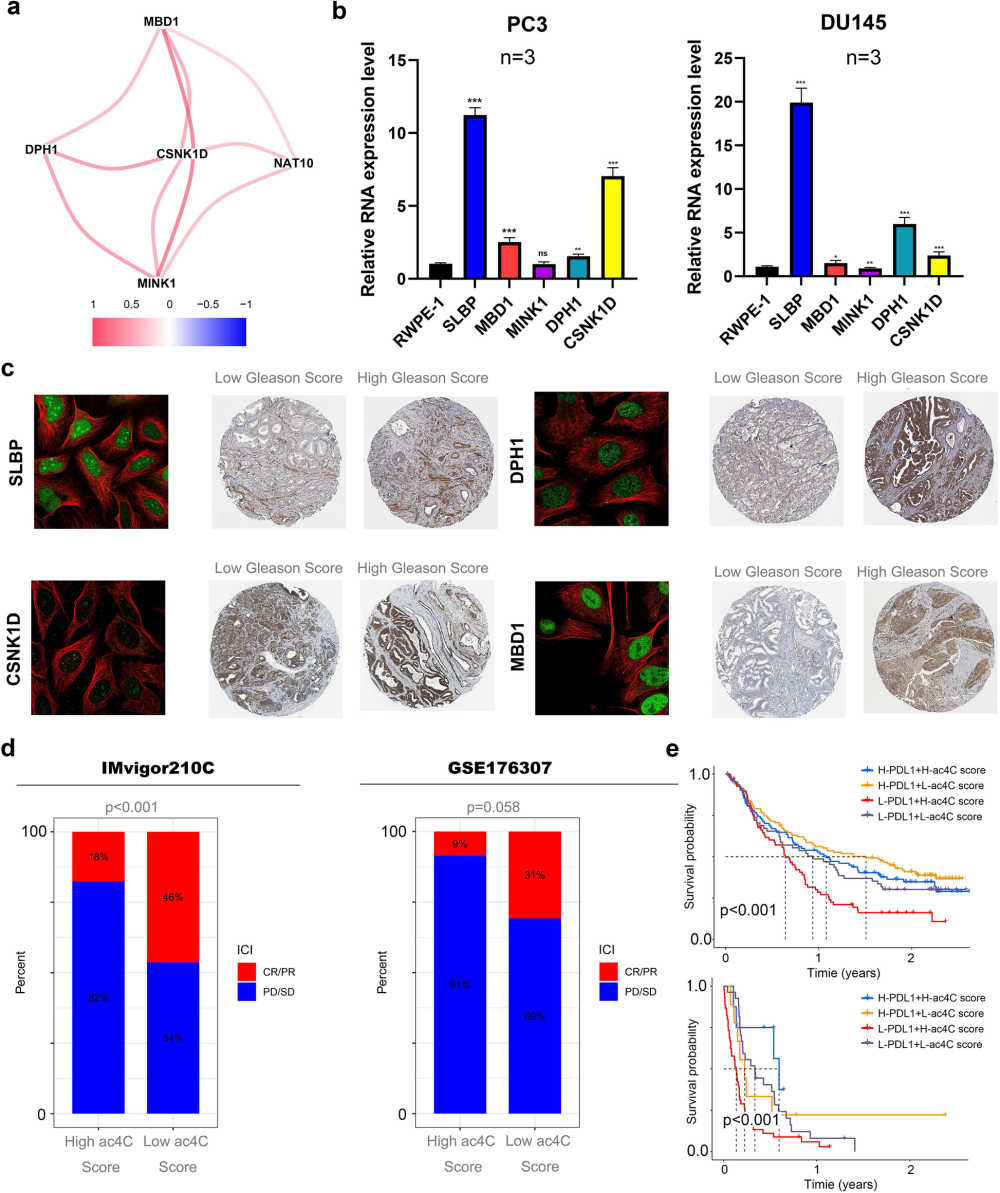
Validation of principal component genes within subtypes from the cellular level. **a** Correlation analysis of principal component genes with NAT10. **b** Validation of principal component gene expression levels in RWPE-1, PC3 and DU145 cell lines. **c** Validation of sublocalization of principal component genes in cells in osteosarcoma cell lines and protein expression in high and low grade samples of prostate cancer (Green represents the target proteins, while red indicates microtubule proteins in the cellular sub-localization images). **d** The proportion of patients responding to immunotherapy in groups with low and high NAT10. **e** The survival curves depict the prognostic outcomes ofl patients stratiflied by high and low expression levels ofl NAT10 and PD-L1

Correlation analysis showed strong associations between NAT10 and genes SLBP, MBD1, MINK1, DPH1, and CSNK1D, emphasizing their interconnected roles (Fig. 8a). To confirm clinical relevance, gene expression of SLBP, MBD1, DPH1, and CSNK1D was assessed in PC3 and DU145 PCa cell lines, showing significantly higher levels than in the normal prostate cell line RWPE-1, indicating potential involvement in cancer progression (Fig. 8b). FISH data confirmed localization in osteosarcoma cells, SLBP, DPH1, and MBD1 are predominantly localized in the nucleus, whereas CSNK1D is primarily located in the cytoplasm, protein expression was notably higher in PCa samples with elevated Gleason scores, linking these genes to aggressive PCa (Fig. 8c). Sankey diagrams illustrated a clear association between ac4C-related subtypes and TCGA-immunized subtypes (Fig. S4a). Drug prediction analysis identified LY2109761, TAF1, and Selumetinib as more effective for patients with low ac4C scores, suggesting targeted therapy advantages for this group (Fig. S4b). Additionally, drug sensitivity analysis indicated that patients with high NAT10 expression were more responsive to standard treatments like docetaxel, cisplatin, and tamoxifen (Fig. S4c). These findings highlight significant correlations between NAT10 and SLBP, MBD1, DPH1, and CSNK1D, which are overexpressed in more aggressive PCa. The data suggests that patients with low ac4C scores may benefit from targeted therapies, while those with high NAT10 expression may respond better to conventional treatments.

### The ac4C score is an important *indicator* of immunotherapy prognosis and efficacy

To evaluate the ac4C score's predictive value for immunotherapy outcomes, validation was conducted using the IMvigor210C and GSE176307 immunotherapy cohorts. Results indicated that patients with lower ac4C scores had significantly higher response rates to immunotherapy compared to those with elevated ac4C expression (Fig. 8d). Additionally, the lower ac4C score group exhibited better clinical outcomes, with prolonged progression-free survival relative to the high ac4C score group (Fig. S5). The predictive power of the ac4C score for PCa prognosis was further validated in the GSE172099 and GSE116918 cohorts, demonstrating consistent generalizability across datasets (Fig. S6a-b). To compare the ac4C score with other prognostic models, three published PCa prognostic models [19–21] were incorporated, and a comparative analysis of AUC values was performed. Findings showed that the ac4C score offered notable advantages in predicting patient prognosis compared to these existing models (Fig. S6c). The relationship between immune markers and ac4C scores was further examined, revealing significant differences in overall survival (OS) between the PD-L1 high-expression group and the high ac4C score group, underscoring the score's relevance to immune-related outcomes (Fig. 8e). Clinical characteristics in patients were analyzed via univariate and multivariate Cox regression analyses (Fig. S7a-b). Based on these insights, a nomogram was developed, integrating NAT10 expression, ac4C score, cisplatin treatment status, and baseline ECOG score to provide a comprehensive risk assessment (Fig. S7c). Calibration curves confirmed the nomogram's accuracy in predicting 1-year, and 1.5-year prognoses for patients (Fig. S7d). Overall, these results highlight the ac4C score as a robust predictor of immunotherapy outcomes and prognosis, with lower scores correlating with enhanced response rates and favorable prognoses. The ac4C score outperforms other prognostic models across various datasets, and the novel nomogram delivers reliable short-term prognosis predictions.

## Discussion

The PCa immune microenvironment, composed of tumor cells, fibroblasts, various immune cells, and a diverse range of cytokines and chemokines, is intricately linked to metastasis, castration resistance, and PCa onset [22, 23]. Understanding post-transcriptional mRNA modifications is vital for grasping tumor development and progression, underscoring the relevance of epitranscriptomics [24, 25]. Among RNA modifications, ac4C modification plays a pivotal role in the processing, distribution, and function of mRNAs [26], warranting further exploration into the role of ac4C acetylation within tumor immune microenvironments. Zhang et al. identified that NAT10 fosters malignant progression in colorectal cancer, closely associating with immune infiltration and immunotherapy outcomes [27], while Liu et al. found that NAT10-mediated ac4C modification is significantly linked with inflammation-associated immune cells, highlighting its critical role in immune regulation [28]. Nonetheless, the role of ac4C in modulating PCa tumor immune environments remains undefined. This study aimed to investigate the correlation between NAT10 expression and PCa cell malignancy, along with elucidating NAT10’s role in the tumor microenvironment. Our findings confirmed, through in vivo and in vitro assays, that NAT10 enhances PCa cell proliferation, invasion, and migration. Additionally, NAT10 was shown to inhibit CD8^+^ T cell accumulation near the tumor via the CCL25/CCR9 axis, fostering an immunosuppressive microenvironment. Subsequently, an ac4C-related immune score derived from multi-database data was developed, which proved effective in prognosticating the outcomes of patients and predicting immunotherapy responses.

This study initially identified a significant overexpression of NAT10 in mCRPC, and further clinical correlation analyses revealed a strong positive association between NAT10 expression and PCa malignancy, including recurrence, drug resistance, and local progression. Previous studies have shown that NAT10 promotes proliferation and invasion in malignancies such as gastric [29], breast [30, 31], and pancreatic cancers [32]. Our in vitro experiments further confirmed that NAT10 regulates PCa cell proliferation, invasion, and migration, aligning with prior findings. Clinical characterization results suggest a robust association between NAT10 and PCa drug resistance and recurrence, further corroborated by existing studies [33, 34], offering new insights into the potential of NAT10-mediated ac4C acetylation as a therapeutic target for overcoming resistance to androgen deprivation therapy in PCa.

The GSEA highlighted NAT10's capacity to influence the PCa tumor microenvironment, specifically via the Wnt and TGF-β signaling pathways, consistent with previous findings [11, 35]. Subsequent multi-database and immune cell infiltration analyses revealed a significant negative correlation between NAT10 expression and the infiltration of cytotoxic immune cells, alongside local immune activation. These observations suggest that NAT10 may facilitate the development of an inhibitory immune microenvironment in PCa. Our findings underscore NAT10’s oncogenic role in promoting glycolysis-immunosuppression crosstalk within cancer cells [11] and show that NAT10-induced NPM1 acetylation enhances PD-L1 transcription and expression, strengthening immune evasion [36]. Previous research has demonstrated the impact of NAT10-mediated ac4C modifications on immune regulation. Li et al. found that ac4C modifications by NAT10 enhanced YWHAH stability in colorectal cancer, contributing to CD8^+^ T cell exhaustion [37]. Chen et al. discovered that NAT10-mediated ac4C acetylation enhances the immunosuppressive function of tumor-infiltrating regulatory T cells (Tregs) through the FOXP1 axis. This modulation of Tregs ultimately contributes to the formation of an immunosuppressive microenvironment [13]. Our results suggest a significant negative correlation between NAT10 expression and CD8^+^ T cell infiltration around the tumor, indicating that NAT10 suppresses the cytotoxic activity of CD8^+^ T cells. Further investigation revealed that NAT10 may reduce CD8^+^ T cell levels by downregulating the expression of the chemokine CCL25, thereby influencing CD8^+^ T cell recruitment. Known as the thymus-expressed chemokine, CCL25 serves as the exclusive ligand for CCR9 [38, 39], a receptor selectively expressed by medullary dendritic cells, thymic cortical epithelial cells, and the small intestinal epithelium under physiological conditions [40]. Notably, recent research indicates that tumor cells can also express CCL25 [41, 42], potentially promoting the infiltration of cytotoxic, CCR9-expressing tumor-infiltrating lymphocytes into the tumor microenvironment, thereby enhancing anticancer effects [43, 44]. Our results also revealed that tumor cells express CCR9, with CCR9 levels significantly upregulated following NAT10 knockdown. This suggests that NAT10 may impede CD8^+^ T cell recruitment by promoting CCR9 expression and competitively binding to CCL25, which disrupts CD8^+^ T cell infiltration and supports the formation of a localized immunosuppressive microenvironment. Thus, these findings propose a mechanism in which NAT10, through modulation of CCL25 and CCR9 dynamics, contributes to immune evasion in PCa by inhibiting CD8^+^ T cell recruitment and activity within the tumor microenvironment.

In this study, a comprehensive screening of immune-related downstream target genes potentially regulated by NAT10 was conducted using WGCNA, incorporating ac4C transcriptional profiles, immune scores, and clinical variables. This analysis facilitated the construction of ac4C score-associated subtypes. The high ac4C score group exhibited a poorer prognosis and increased cytotoxic immune cell infiltration within the tumor microenvironment. Key genes identified in this subtype included SLBP, MBD1, MINK1, DPH1, and CSNK1D, which are noted in existing literature for their roles in tumor malignancy and progression [45–48], thereby reinforcing the reliability of the ac4C score. To validate the ac4C score's applicability in evaluating the immune microenvironment, two immunotherapy datasets and two PCa datasets were incorporated. Results confirmed that patients with lower ac4C scores had better prognoses and improved immunotherapy responses compared to those with higher scores, underscoring the ac4C score's effectiveness in immune microenvironment assessment in PCa.

This study, however, has certain limitations. Specifically, while findings suggest that the CCL25/CCR9 axis is a key element in the immunosuppressive microenvironment of PCa and likely regulated by NAT10, additional molecular evidence is necessary to definitively confirm whether NAT10 influences CCL25 and CCR9 expression through the ac4C acetylation pathway. Furthermore, the roles of the principal genes within the ac4C score in PCa require additional investigation to validate their clinical implications. Expanded studies are also needed to fully establish the ac4C score’s capacity for assessing local immune characteristics and predicting immunotherapy outcomes in patients with PCa.

In conclusion, this research offers an in-depth view of NAT10’s role in enhancing PCa cell proliferation and infiltration both in vitro and in vivo. It demonstrates that NAT10 may restrict CD8^+^ T cell recruitment via modulation of CCL25, thereby fostering an immunosuppressive environment that supports PCa progression. Additionally, the ac4C score, a marker derivative of NAT10, shows promise as a robust prognostic indicator for immunotherapy responsiveness in PCa, providing a potentially more precise assessment of patient outcomes.

## Materials and methods

### Data cohorts

Transcriptomic and proteomic data were sourced from public repositories, encompassing 499 tumor samples and 52 normal samples. Additionally, data from the TCGA-mCRPC group, including 91 mCRPC samples, were accessed via The Cancer Genome Atlas (TCGA) database (https://cancergenome.nih.gov/). The ICGC-PRAD dataset was obtained from the ICGC Data Portal (https://dcc.icgc.org), and clinical data for these samples were sourced from the UCSC Xena platform. The IMvigor210 cohort was accessed through http://research.pub.com/imvigor210corebiologies/, while the GSE176307 immunotherapy cohort was acquired from the Gene Expression Omnibus (GEO) database (https://www.ncbi.nlm.nih.gov/geo). Incomplete data entries were excluded from the study. For classification, PCa samples with a clinical stage of T0M0N0, no biochemical relapse, and complete response to initial and subsequent treatments were classified as non-mCRPC. Additionally, protein organization data, single-cell sequencing data, and select IHC images were retrieved from the Human Protein Atlas (HPA) database (https://www.proteinatlas.org). The mRNAsi index for TCGA-derived PCa samples was sourced from Malta et al*.* [49].

### Clinical samples from our center

Clinical samples were obtained from patients diagnosed with PCa, confirmed by pathologists, and treated with radical prostatectomy at Shanghai Tenth People's Hospital (STPH) between August 2020 and April 2022.

### Weighted gene co-expression network approach

WGCNA was utilized to identify molecules associated with the immune microenvironment and malignant progression in PCa. Using the R package "WGCNA", a co-expression network was constructed to evaluate the correlations between gene modules and clinical features. The module with the strongest correlation to targeted clinical indicators (*p* < 0.05) was selected for further analysis.

### Consensus cluster

Based on mRNA expression profiles from patients with TCGA-PRAD, unsupervised consensus clustering was performed using the "consensusClusterPlus" R package, with clusters validated across 100 replicates and a pltem value of 0.8. An additional non-supervised clustering analysis was conducted on the metadata set using the "NMF" package, with an optimal coexistence correlation coefficient of k = 2 to define cluster types. PCA was then applied to assess the ability of molecular clusters to distinguish between subtypes and to construct the ac4C Score, enabling further analysis of molecular subtype differentiation. The formula is presented as follows:

$$ac4C Score=\sum_{i}^{n}\text{PCs}$$

In this formula, S represents the expression level of principal component genes associated with ac4C subtypes.

### Cell lines

Cell lines for normal prostate epithelial (RWPE-1) and PCa (VCaP, C4-2, PC3, DU145, LNCaP, 22RV1 and RM-1) were sourced from the Chinese Academy of Sciences. Human derived PCa cells were cultured in RPMI-1640 supplemented with 1% penicillin–streptomycin and 10% fetal bovine serum (Gibco, USA). The murine prostate cancer cell line RM-1 was cultured in DMEM supplemented with 10% fetal bovine serum (Gibco, USA) and 1% penicillin–streptomycin. For RWPE-1 cells, 1% Keratinocyte Growth Supplement was added to Keratinocyte-SFM (Invitrogen, USA). All cells were incubated at 37 °C with 5% CO_2_. siRNA and shRNA sequences used in experiments are listed in Table S2.

### Flow cytometry analysis of T cell-mediated tumor killing

Tumor cells were seeded in a 12-well plate at a density of 1 × 10^5^ cells per 1.5 mL and allowed to adhere. T cells were subsequently added at a 1:1 effector-to-target ratio, and the volume was adjusted to 2.5 mL. After 24 h, suspended effector cells were harvested by washing with PBS, followed by centrifugation at 1800 rpm for 5 min to obtain the cell pellet. Tumor cells were similarly collected by centrifugation at 800 rpm for 5 min. The collected cells were resuspended in 50 μL PBS per tube, and T cell surface marker CD3 was stained using an APC-conjugated antibody in 50 μL PBS. GFP content was used as an indicator of remaining tumor cells. Cells were stained at 4 °C for 30 min, resuspended in 1 mL PBS, and centrifuged. The resulting cell pellet was fixed in 1% paraformaldehyde and subjected to flow cytometry analysis.

### CD8^+^ T cell Transwell assay

For the Transwell migration assay, low-adhesion 24-well plates with 5 μm pore inserts were pre-equilibrated by adding 200 μL of blank culture medium to the upper chamber and 600 μL to the lower chamber, followed by incubation at 37 °C for 1–2 h. CD8^+^ T cells were prepared by centrifugation, counted, and then diluted to a concentration of 5 × 10^7^ cells/mL in basal culture medium. The medium in the inserts was removed, 200 μL of the T cell suspension was added to the upper chamber, and tumor cell supernatants from different experimental groups were added to the lower chamber. Plates were incubated at 37 °C for 2 h. After incubation, the upper chamber was carefully removed, and cells in the lower chamber were collected, transferred to an EP tube, centrifuged at 1800 rpm for 5 min, and stained with 20 μL of crystal violet. Following a 30-min staining period, 5 μL of the stained cell suspension was placed on a glass slide, covered with a coverslip, and imaged under a microscope.

### 3D-Transwell invasion assay

For the 3D-Transwell invasion assay, inserts were pre-coated with Matrigel to simulate the extracellular matrix environment. Matrigel was diluted in serum-free medium, added to the inserts, and allowed to solidify at 37 °C. Cells were harvested and resuspended in a serum-free medium at a density of 1 × 10^5^ cells/mL, and 100–200 μL of the cell suspension was added to the upper chamber of the inserts. The lower chamber was filled with a medium containing 10% FBS as a chemoattractant. After 48 h of incubation at 37 °C with 5% CO_2_, non-invading cells on the upper side of the membrane were removed, while invaded cells on the lower surface were fixed with 4% paraformaldehyde, stained with crystal violet, and quantified under a microscope. The number of invaded cells was analyzed across experimental groups to assess the invasion potential under various conditions.

### Organoids

Prostate tumor tissue was collected through radical prostatectomy and cut into 1–2 mm fragments. These tissue pieces were then enzymatically digested for 1 h in a solution containing type IV collagenase (1 mg/mL, 40510ES76, Yeason) and Y-27632 (10 μM, HY-10071, MCE). After digestion, the tissue was transferred to pre-warmed 24-well plates pre-coated with Basement Membrane Extract (BME, 3533–001–02) to provide structural support. Following solidification of the BME, human prostate cancer organoids were introduced and cultured further.

### Data analysis

Data analyses of public datasets were performed using R (Version 4.2.2), and experimental data analyses were conducted in GraphPad Prism 8. To assess differences in immune cell types, TIDE scores, and immune checkpoints between groups, both Wilcoxon and t-tests were employed. Statistical significance was defined as p-values less than 0.05.

## Supplementary Information

Supplementary Material 1.

Supplementary Material 2.

## Data Availability

The sources of the data and materials used in this study are detailed within the manuscript. Additional information is available upon request from the corresponding author.
