# Supplementary figures and images for: Acetyltransferase NAT10 promotes an immunosuppressive microenvironment by modulating CD8+ T cell activity in prostate cancer
Source: Mol Biomed. 2024 Dec 9;5:67. doi: 10.1186/s43556-024-00228-5 (PMC11625704; doi:10.1186/s43556-024-00228-5)

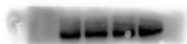

**GAPDH 37kDa**

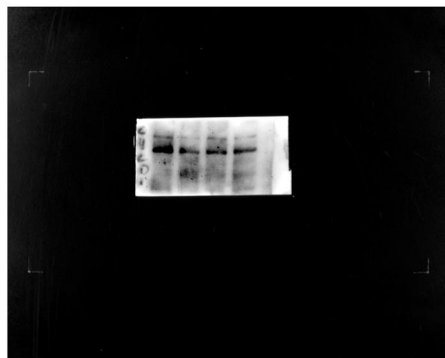

**CyclinD1 36 kDa**

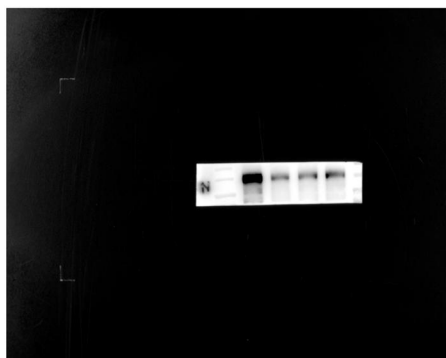

**NAT10 116 kDa**

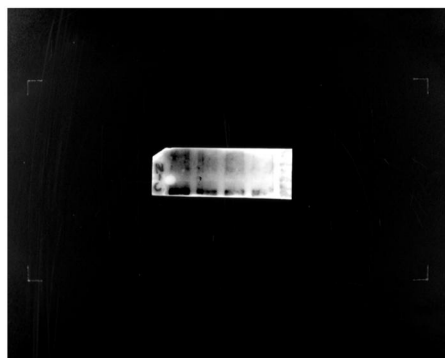

**N-cadherin 120 kDa**

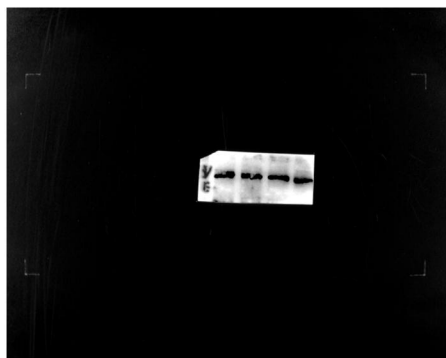

**Vimentin 57 kDa**

Supplement: Supplementary file 1 — Supplementary Material 1. [file 43556_2024_228_MOESM1_ESM.pdf]
