# Supplementary material for: Acetyltransferase NAT10 promotes an immunosuppressive microenvironment by modulating CD8+ T cell activity in prostate cancer
Source: Mol Biomed. 2024 Dec 9;5:67. doi: 10.1186/s43556-024-00228-5 (PMC11625704; doi:10.1186/s43556-024-00228-5)
Supplement: Supplementary file 2 — Supplementary Material 2. [file 43556_2024_228_MOESM2_ESM.docx]

**NAT10 Promotes an Immunosuppressive Microenvironment by Modulating CD8^+^ T Cell Activity in Prostate Cancer**

**Ji Liu^1,2#^, Zhuoran Gu^1,2#^, Libin Zou^1,2#^, Zhijin Zhang^1,2^, Liliang Shen^4^, Ruiliang Wang^1,2^, Shaobo Xue^3^, Jiang Geng^1,2^, Shiyu Mao^1,2*^, Wentao Zhang^1,2*^, Xudong Yao^1,2*^**

1 Department of Urology, Shanghai Tenth People's Hospital, School of Medicine, Tongji University, Shanghai, China;

2 Institute of Urinary Oncology, School of MedicineTongji, University, Shanghai, China;

3 Department of Central Laboratory, Clinical Medicine Scientific and Technical Innovation Park, Shanghai Tenth People's Hospital, Shanghai, 200435, China.

4 Department of Urology, the Affiliated People's Hospital of Ningbo University,251 East Baizhang Road, Ningbo City, Zhejiang Province, China

# These authors contributed equally to this work.

* Correspondence:

Corresponding Author: Prof. Xudong Yao; Prof. Wentao Zhang; Prof. Shiyu Mao

Email address:

[yaoxudong1967@163.com;](mailto:email@uni.eduyaoxudong1967@163.com;) zhangwentao98@163.com; [maoshiyu1144@sina.com](mailto:maoshiyu1144@sina.com;)

**Supplementary Figures**

**
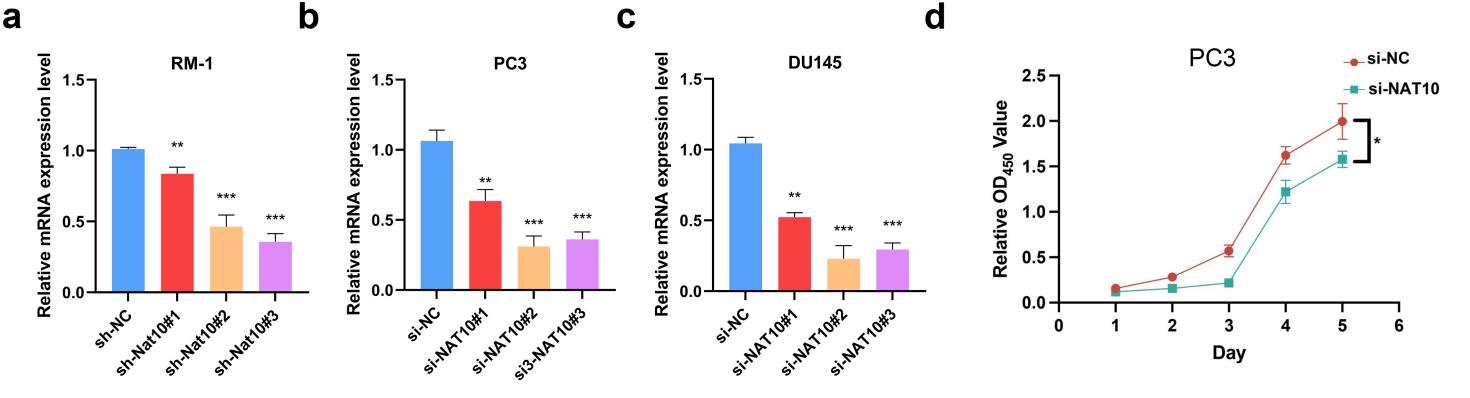
**

**Figure S1.** (a-c) Validation of the efficiency of siRNA and shRNA in various cell lines. (d) Effect of knockdown of NAT10 on proliferative capacity of PC3 cells assessed by CCK8 assay

**
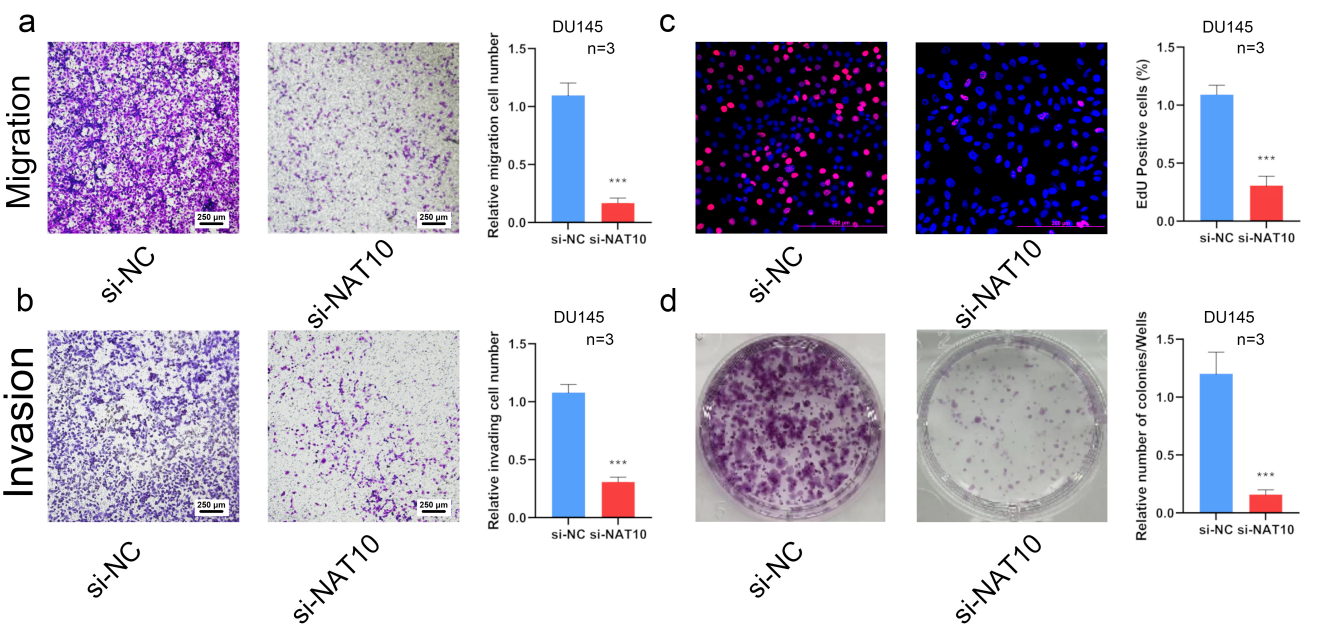
**

**Figure S2. The effect of knockdown of NAT10 on the proliferative capacity of DU145 cells was assessed by plate cloning, Transwell and EdU assays.** (a-b) Transwell assays were conducted to assess the changes in migration and invasion abilities of the prostate cancer cell line DU145 after NAT10 knockdown. (c-d) Plate cloning and EdU assays were performed to evaluate the changes in proliferation capacity of DU145 cells following NAT10 knockdown. (*P < 0.05; **P < 0.01; ***P < 0.001)

**
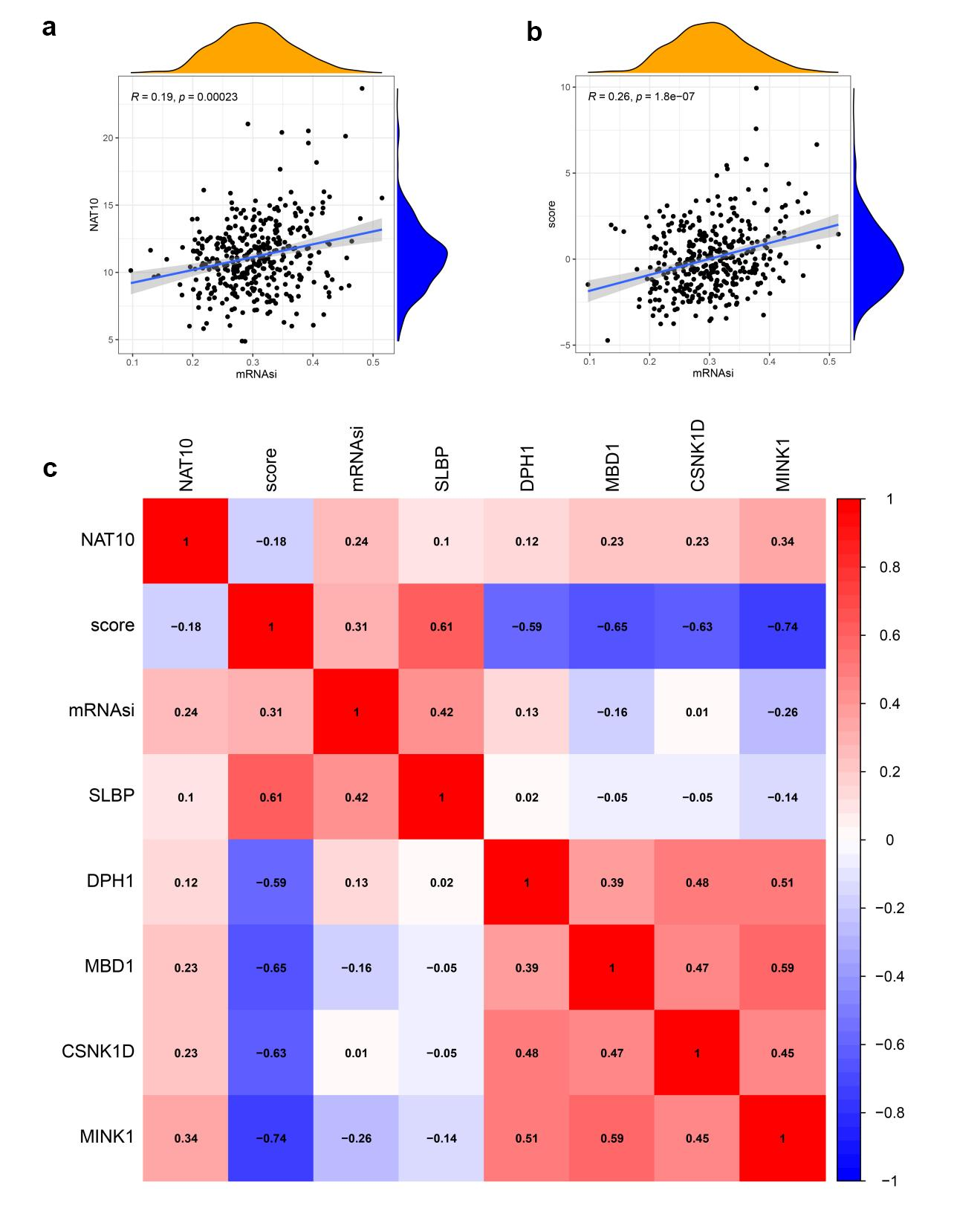
**

**Figure S3. Validation of the correlation between ac4C score and tumor stem cells** (a-b) Correlation analysis to explore the correlation between ac4C score, NAT10 and mRNAsi. (c) Exploring the correlation between principal component genes and NAT10 and mRNAsi.

**
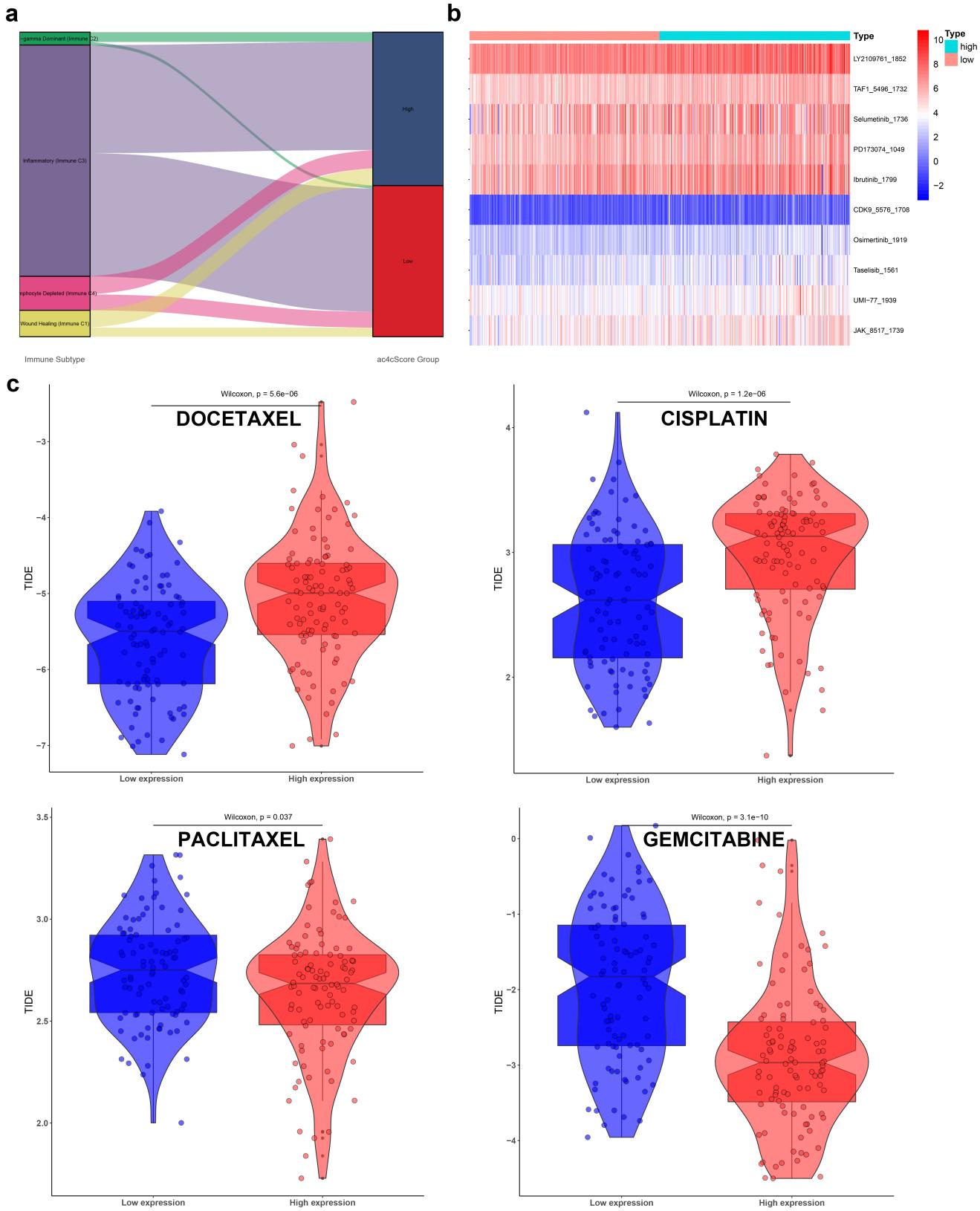
**

**Figure S4. Exploring the correlation between ac4C scores and drug sensitivity.** (a) Exploring the correlation between ac4C subtypes and TCGA immune subtypes. (b) Sensitivity analysis for drugs between NAT10 high and low expression groups. (c) Sensitivity analysis between NAT10 high expression and low expression groups for clinically used chemotherapeutic agents.

**
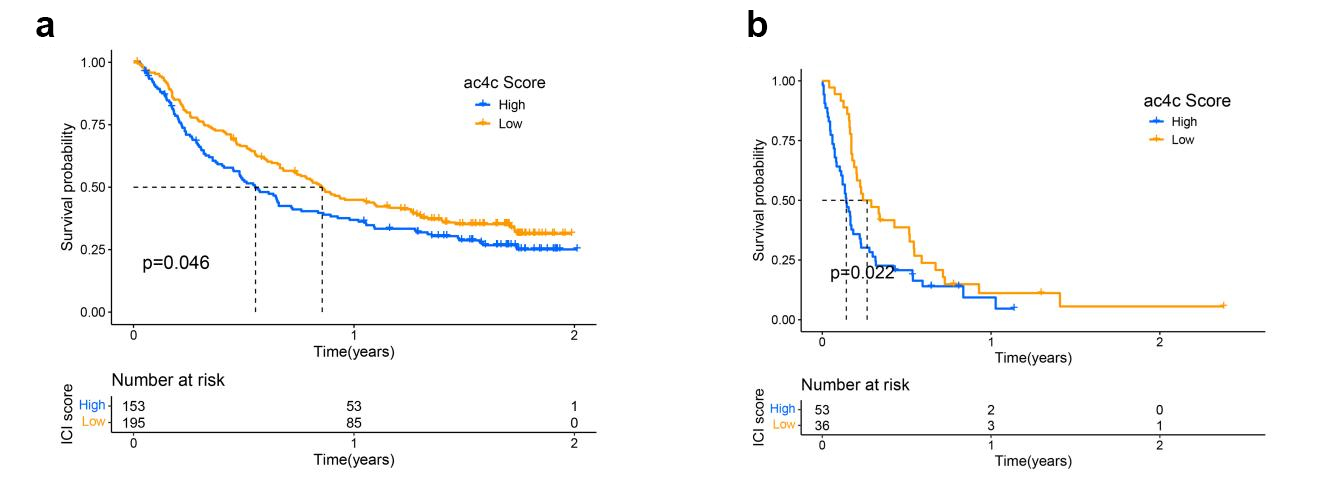
**

**Figure S5. Exploring the ability of ac4C scores to predict prognosis in immunotherapy.** (a) Correlation of ac4C score with patient prognosis in the IMvigor210C dataset. (b) Correlation of ac4C score with patient prognosis in the GSE176307 dataset.


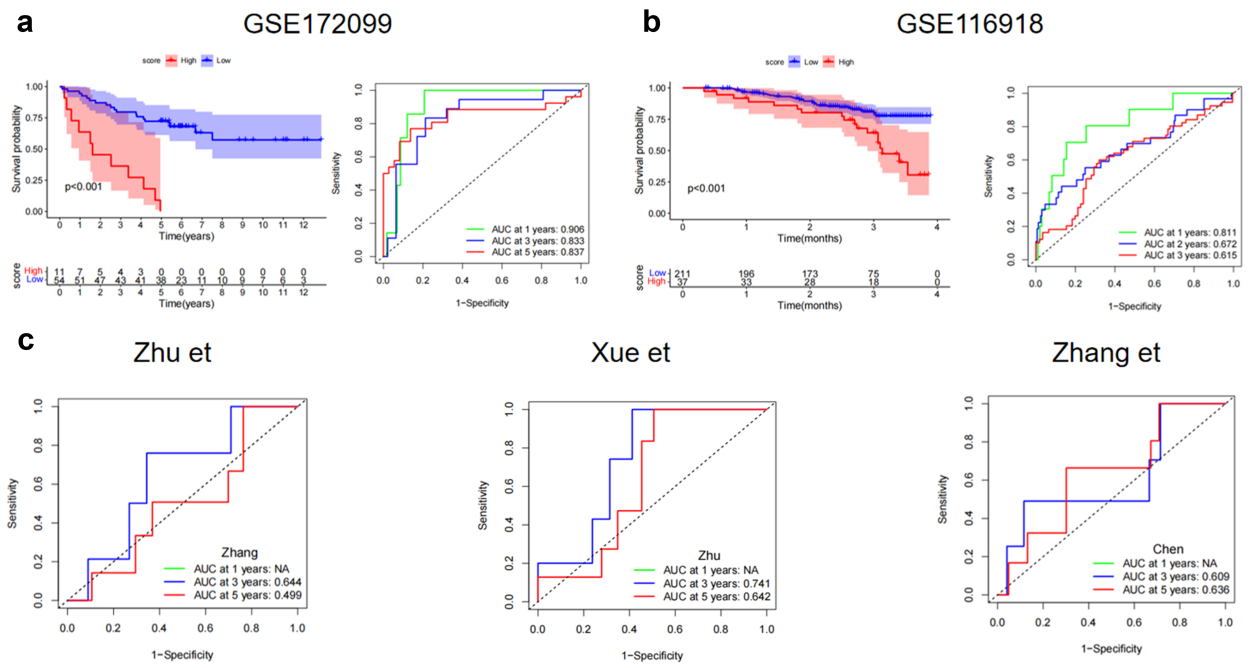


**Figure S6. Exploring the ability of ac4C scores to predict prognosis in others external database**  (a-b) Correlation of ac4C score with patient prognosis in the GSE172099 and GSE116918 dataset. (c) To investigate the efficacy of published prognostic prediction models for prostate cancer in this study cohort.


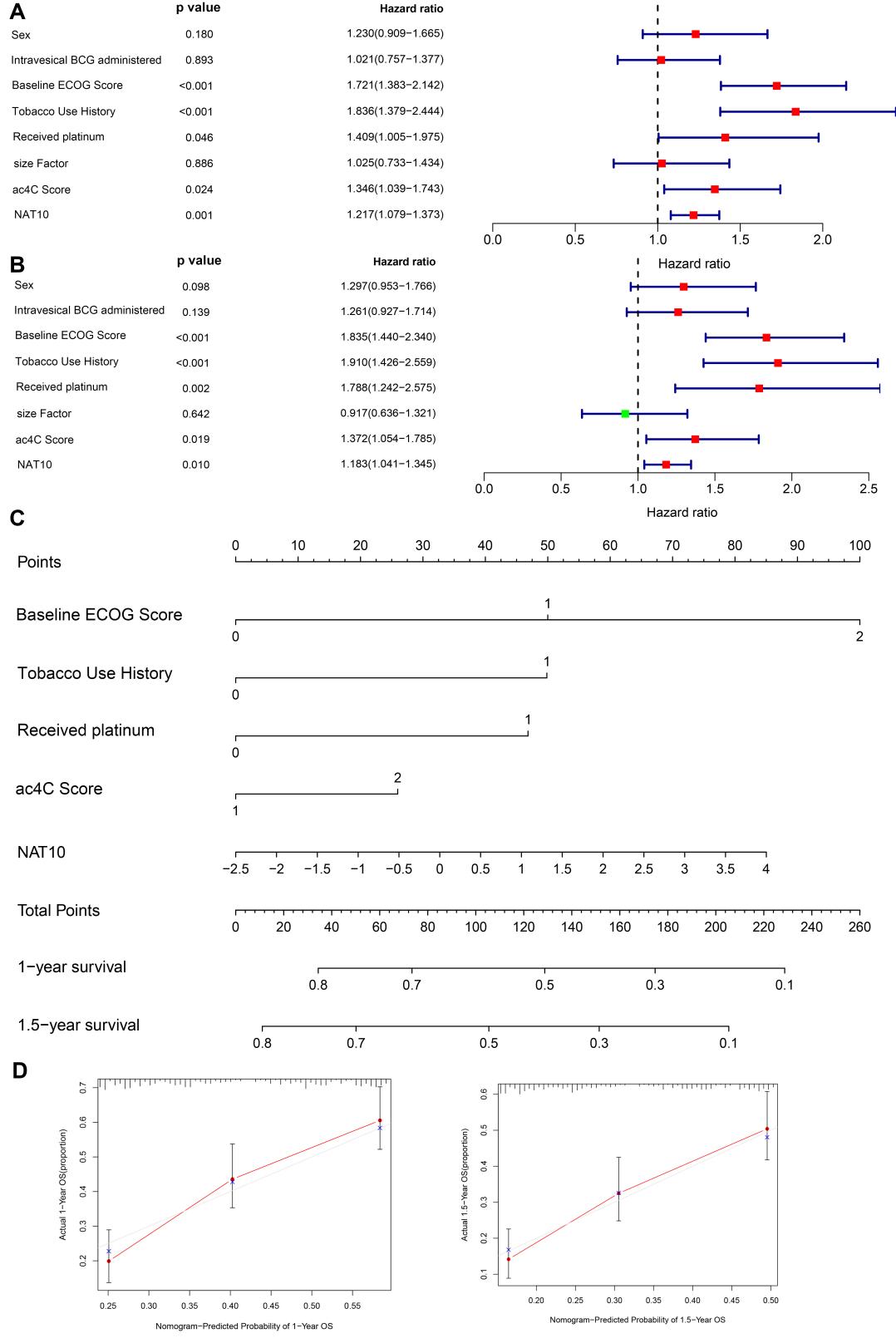


**Figure S7. Construction of nomogram to Predict the Prognosis of PCa Patients undergo immunity therapy** (a-b) Unicox analysis of patient clinical characteristics in IMvigor210C cohort. (c) The Nomogram constructed by combining ac4C score gourp with clinical characteristics predicts the prognosis of patients who accepted ICB in IMvigor210C. (d) The calibration and DCA curve of the nomogram.

**Supplementary Tables**

**Table S1: The sequence of Primer**

| Gene | Sequence |
| --- | --- |
| Human-NAT10 | Forward 5‘-AACGAGCTGGATTTGTTCCTG-3’ |
|  | Reverse 5’-CTGGTCAGCCTCATCCTCATC-3’ |
| Mouse-Nat10 | Forward 5‘-ATGACCTGCTGTGCCTGGATTG |
|  | Reverse 5’-GCTGGAGGAAAACTTCAGAGGC |
| Human-SLBP1 | Forward 5‘-CAAGACACCTTCGACAACCT-3’ |
|  | Reverse 5’-GTCCCATGAACGTCGACTATAC-3’ |
| Human-MBD1 | Forward 5‘-CCCTGCGTGTGATCTCACC-3’ |
|  | Reverse 5’-ACCTGACGTTTCCGAGTCTTG-3’ |
| Human-MINK1 | Forward 5‘-CATCTGCAGGGAGATTCTCAGG-3’ |
|  | Reverse 5’-CTGTAGTCATAGGTGGCATCGG-3’ |
| Human-DPH1 | Forward 5‘-GGTATGAATGACTAATAGGGATGCC-3’ |
|  | Reverse 5’-TGTCATATAGGTACAGCGCT-3’ |
| Human-CSNK1D | Forward 5‘-CAGGAGAAGAGGTTGCCATCA-3’ |
|  | Reverse 5’-CAAGCAGCAGGACGGTTTTG-3’ |
| Human-CCL25 | Forward 5‘-CCAAGGTGCCTTTGAAGACT-3’ |
|  | Reverse 5’-TCCTCCAGCTGGTGGTTACT-3’ |
| Human-CCR9 | Forward 5‘-ATGTCAGGCAGTTTGCGAG-3’ |
|  | Reverse 5’-TGCAGTACCAGTAGACAAGGAT-3’ |
| Human-GAPDH | Forward 5‘-GAGCGAGATCCCTCCAAAAT-3’ |
|  | Reverse 5’-GGCTGTTGTCATACTTCTCATGG-3’ |
| Mouse-Gapdh | Forward 5‘-CATCACTGCCACCCAGAAGACTG-3’ |
|  | Reverse 5’-ATGCCAGTGAGCTTCCCGTTCAG-3’ |

**Table S2: The sequence of si-RNA and sh-RNA**

| Name | Sequence |
| --- | --- |
| Human-si-NAT10#1 | 5’-GGCCAAAGCUGUCUUGAAAUU-3’ |
| Human-si-NAT10#2 | 5’-GGACCAUGAACUCACUCAAGC-3’ |
| Human-si-NAT10#3 | 5’-CAGCACCACUGCUGAGAAUAAGAUU-3’ |
| Mouse_sh-Nat10#1 | 5’-GGCCAAAGCTGTCTTGAAATT-3’ |
| Mouse_sh-Nat10#2 | 5’-CTGTTTGAATTTGTATTTAAAGG-3’ |
| Mouse_sh-Nat10#3 | 5’-TGGAAACGAGCTGGATTTGTTCC-3’ |

**Table S3: The detail of antibody**

| Name | Brand | Number |
| --- | --- | --- |
| anti-mouse CD4 APC | BioLegend | Cat#100411 |
| anti-mouse CD8a PE | BioLegend | Cat#100707 |
| anti-mouse CD86 APC | BioLegend | Cat#105011 |
| anti-human CCR9 APC | BioLegend | Cat#358907 |
| anti-mouse CD8a FITC | BioLegend | Cat#300905 |
| GAPDH | Abcam | ab8245 |
| NAT10 | proteintech | No. 13365-1-AP |
| 1. Cadherin | Abcam | ab76011 |
| Vimentin | Abcam | ab92547 |
| CyclinD1 | Abcam | ab16663 |
| anti-human CD8a | Abcam | ab237709 |

**Supplementary Methods**

**Identification of mRNAs with differential expression**

The EDGER R package, available at http://bioconductor.org/package/EDGER/, was employed to identify differentially expressed mRNAs and proteins within the TCGA-ICGC-PRAD dataset. The screening criterion utilized was a false discovery rate (FDR) of less than 0.05. Subsequently, a Venn diagram was utilized to select molecules associated with the tumor immune microenvironment and malignant progression.

**Enrichment Analysis**

Biological pathways between clusters were assessed by gene set variation analysis (GSVA) and gene set enrichment analysis (GSEA). GO (Gene Ontology) describes biological processes, molecular functions, and cellular components of genes. The Kyoto Encyclopedia of Genes and Genomes (KEGG) were used to predict the gene pathway way involved in NAT10.

**Real time quantitative PCR (RT-qPCR)**

The extraction of total RNA from cells was performed in accordance with the instructions provided by the manufacturer, using TRIzol® reagent (Thermo Fisher Scientific, Inc, USA). RT-qPCR analysis was conducted using Hieff® qPCR SYBR Green Master Mix (11203ES03, Yeasen Biotechnology; Shanghai, China) on the 7900HT Fast Real-Time PCR System, following the recommended protocols. The expression levels of relative mRNA were normalized using GAPDH as a control. The primer sequences used are provided in Table S1.

**Immunohistochemical staining**

Tissue sections undergo deparaffinization and rehydration through a series of alcohol gradients. Antigen retrieval is then performed, typically using a high-pH targeted retrieval solution and heat-induced epitope retrieval (HIER) to unmask antigenic sites. To block endogenous peroxidase activity, tissue sections are treated with 3% hydrogen peroxide for 20 minutes. After washing, primary antibodies are incubated overnight at 4°C. The next day, sections are washed and incubated with HRP-conjugated secondary antibodies at room temperature for 1 hour. A chromogenic substrate, such as DAB, is added to visualize the antibody-antigen complex, producing a brown precipitate at the antigen site. Finally, sections are counterstained with hematoxylin for contrast, mounted, and examined under a microscope to assess the intensity and distribution of the staining.

**Migration and invasion assay**

This experiment was performed in transwell plate with multipolar 8.0 μl polycarbonate membrane (Corning, USA). Cells with mixed with serum free medium and kept in upper chamber. And 500 μl of complete medium was added to lower chamber. Then cells were incubated at 37°C with 5% CO_2_. After 48 hours cells with stained with crystal violet and photos taken using 100X magnification. Each experiment was performed in triplicate.

**Western blot**

Total protein was extracted using SDS lysis buffer (Beyotime, Nantong, China) and quantified with the BCA Protein Assay Kit (Beyotime, Nantong, China). Protein samples (40 µL per lane) were loaded onto a 10% SDS-PAGE gel. The proteins were then transferred to polyvinylidene fluoride (PVDF) membranes (Millipore, Burlington, MA, USA). Non-specific binding was blocked with 5% milk. Primary antibodies were applied and incubated overnight at 4°C. The following day, membranes were washed three times for 10 minutes each with PBST. Secondary antibodies were applied and incubated for 1 hour at room temperature. The membranes were then washed three times for 10 minutes each with PBST. Protein bands were visualized using a silver stain detection system (Beyotime, Nantong, China). The antibodies used in this procedure are listed in Table S3.

**Nomogram development and validation**

Univariate and multivariate Cox regression analyses were used to construct nomograms to predict the impact of target genes and other clinicopathological features on prognosis and metastasis in PCa patients. The Nomogram was validated by the calibration plots and consistency index (C-index) using the rms package v5.1 in R (https://cran.r-project.org/web/packages/rms /index.html).

**Animal model**

The animal experiments were approved by the Animal Care and Use Committee of Shanghai Tenth People’s Hospital, Tongji University (approval number: SHDSYY-2021-3028). Sixteen 6-week-old male C57 mice were used to establish a subcutaneous xenograft model. These mice were subcutaneously injected with 1 × 10^6^ RM-1-NC or RM-1-sh-NAT10 cells. Tumor size was measured every three days. Tumor volume was calculated using the formula: 0.5 × length × width², and tumors were weighed after three weeks. A portion of the tumor tissues was used for flow cytometry analysis. The antibodies used for this model are listed in Table S3.

**Immune cell profiling by flow cytometry**

To generate single-cell suspensions from xenografted murine prostate cancer (PCa) tumors, 5 mL of collagenase IV (1 mg/mL, 40510ES76, Yeason) and 5 µg/mL of DNAse I (Beyotime, D7076) were mixed with 5 mL of cell culture medium and incubated for 1 hour. After incubation, the cells were labeled with Live/Dead dye (BioLegend, Zombie UV Fixable Viability Kit) at a 1:1000 dilution in PBS on ice for 20 minutes, protected from light. Next, cell surface markers were stained with specific antibodies on ice for 30 minutes. The cells were then analyzed by flow cytometry using the BD LSRFortessa X-20 system, and FlowJo software was used for data analysis. The antibodies used are detailed in Table S3.

**Evaluation of the level of immune infiltration**

Using the transcriptional expression profiles of the TCGA-PRAD and ICGC-PRAD cohorts, scores related to immune activity, estimated tumor score, and tumor purity were calculated with "ESTIMATE" and "ssGSEA" (R package). The MCPcounter tool validated immune cell infiltration, while CIBERSORT deconvolution assessed immune response activation, infiltration of specific immune cell subtypes, and immunocyte composition in PCa samples. Additionally, expression levels of PD-L1 and CTLA-4 were analyzed across groups to evaluate immunotherapy efficacy.

**Isolation of PBMCs and Preparation of T Cells**

For immune cell preparation, ficoll lymphocyte separation medium, saline, and *X-VIVO* complete medium were pre-warmed to 37°C. Fresh peripheral blood underwent centrifugation at 1800 rpm for 10 minutes to remove plasma. The blood was then diluted 1:1 with saline to create a blood cell suspension, which was carefully layered over Ficoll in a sterile centrifuge tube (1:2 Ficoll: blood cell suspension ratio) to form a distinct interface. This tube was centrifuged at 1000 g for 20 minutes with slow acceleration and no brake. Post-centrifugation, four layers were observed: plasma, PBMCs, Ficoll, and red blood cells. The PBMC layer was aspirated and transferred to a new tube, washed twice with saline, and centrifuged at 1800 rpm for 5 minutes each time. Cells were then treated with 1 × RBC lysis buffer for 7 minutes at room temperature, shielded from light. After stopping the lysis with saline, cells were resuspended in *X-VIVO* complete medium, counted, and incubated at 37°C with 5% CO_2_. For T cell activation, 24-well plates were pre-coated with aCD3 and aCD28 antibodies (1 µg/mL in PBS) the day before PBMC isolation to provide co-stimulatory signals. Plates were incubated overnight at 4°C, then freshly isolated PBMCs were plated at a density of 3-10 × 10^5^ cells/mL in 1.5 mL per well and cultured under

**Enzyme-Linked Immunosorbent Assay (ELISA)**

CCL25 levels were quantified using an ELISA kit (Laier Bio, LE-H2207, China) according to the manufacturer's instructions. Samples and standards were added to a 96-well microplate pre-coated with a monoclonal antibody specific for human CCL25. After a 2-hour incubation at room temperature, unbound substances were removed by washing with wash buffer.A biotin-conjugated anti-CCL25 detection antibody was added, followed by a 1-hour incubation at room temperature. Following additional washes, a streptavidin-HRP conjugate was applied, and incubation continued for 30 minutes. After a final wash, tetramethylbenzidine substrate was added, and the plate was incubated in the dark at room temperature for 15 minutes to allow color development. The reaction was stopped with 2N sulfuric acid, and optical density (OD) readings were measured at 450 nm using a microplate reader (Model, Manufacturer, City, Country). A standard curve was constructed by plotting absorbance values of known recombinant CCL25 concentrations against their concentrations. Sample CCL25 concentrations were determined by interpolating from this curve. Each sample was analyzed in duplicate, with mean values reported.
